# Engineering Performance, Environmental and Economic Assessment of Pavement Reconstruction Using Cold In-Place Recycling with Foamed Bitumen: A Municipal Road Case Study

**DOI:** 10.3390/ma19010083

**Published:** 2025-12-25

**Authors:** Justyna Stępień, Anna Chomicz-Kowalska, Krzysztof Maciejewski, Patrycja Wąsik

**Affiliations:** 1 Department of Transportation Engineering, Faculty of Civil Engineering and Architecture, Kielce University of Technology, al. Tysiąclecia Państwa Polskiego 7, 25-314 Kielce, Poland; justynas@tu.kielce.pl (J.S.); kmaciejewski@tu.kielce.pl (K.M.); 2 Faculty of Civil Engineering and Architecture, Kielce University of Technology, al. Tysiąclecia Państwa Polskiego 7, 25-314 Kielce, Poland; patrycjawasik322@gmail.com

**Keywords:** pavement rehabilitation, reclaimed asphalt pavement (RAP), reclaimed aggregate (RA), cold in-place recycling (CIR), foamed bitumen (FB), sustainability technologies

## Abstract

**Highlights:**

**What are the main findings?**

- CIR-FB design increased fatigue life by approximately three to twenty times at equal thickness.
- About 92% of pavement was reclaimed and reused in situ, and virgin material demand fell by approximately 27%.
- Demolition waste decreased by approximately 39% and transport demand was reduced by a factor of five to six.
- The carbon footprint was approximately 16% lower, and the construction-stage cost was reduced by approximately 19%.
- Higher FB/C stabilizer content increased stiffness and fatigue resistance, but also increased embodied emissions, confirming the need for balanced stabilizer dosage.

**What is the implication of the main finding?**

- CIR-FB supports the implementation of circular economy principles in municipal road networks.
- The method enables material conservation and reduces environmental impact.
- The approach decreases fuel consumption, transport intensity and binder consumption.
- The framework is scalable for low- and medium-traffic municipal applications.
- The findings support policy goals related to GHG emission reduction and resource efficiency objectives.

**Abstract:**

Modernizing municipal roads requires rehabilitation strategies that ensure adequate structural performance while reducing environmental and economic burdens. Although cold in-place recycling with foamed bitumen (CIR-FB) has been widely investigated, integrated assessments combining mechanistic–empirical modeling with LCA and LCCA remain limited—particularly for municipal roads in Central and Eastern Europe, where reclaimed asphalt pavement (RAP) quality, climatic conditions and budget constraints differ from commonly studied regions. This study compares two reconstruction variants for a 1 km road section: a conventional design using virgin materials (V_1_-N) and a recycling-based alternative (V2-Rc) incorporating RAP from the existing wearing and binder layers and reclaimed aggregate (RA) from the existing base. CIR-FB mixture testing (stiffness ≈ 5.25 GPa; foamed bitumen = 2.5%, cement = 2.0%) was integrated into mechanistic–empirical fatigue analysis, material-flow quantification, LCA and LCCA. The V_2_-Rc variant achieved a 3–21-fold increase in fatigue life compared to V_1_-N at equal thickness. Material demand decreased by approximately 27%, demolition waste by approximately 39%, and approximately 92% of the existing pavement was reused in situ. Transport work was reduced five-fold (veh-km) and more than six-fold (t-km). LCA showed a 15.9% reduction in CO_2_-eq emissions, while LCCA indicated approximately 19% lower construction cost, with advantages remaining robust under ±20% sensitivity. The results demonstrate that CIR-FB, when supported by proper RAP/RA characterization, can substantially improve structural, environmental and economic performance in municipal road rehabilitation.

## 1. Introduction

### 1.1. Background

The maintenance and modernization of road infrastructure represent fundamental drivers of socio-economic development, as efficient and reliable transport networks determine mobility, economic accessibility, and regional competitiveness. In the context of steadily increasing traffic volumes and the progressive deterioration of pavement conditions, the implementation of advanced rehabilitation technologies has become imperative. Such technologies are expected not only to ensure high structural and engineering performance but also to minimize adverse environmental impacts. Recent research emphasizes that the integration of engineering performance, economic, and environmental dimensions into road maintenance strategies is fully consistent with the principles of the circular economy and the climate policy framework of the European Union [1,2]. This assertion is supported by comprehensive evaluations carried out by European national road authorities as well as comparative assessments of Member States’ progress in implementing circular economy practices [3,4].

Environmental considerations have become increasingly significant in pavement design and construction. The European Union regulations on greenhouse gas (GHG) emission reduction and circular economy strategies drive the adoption of technologies that mitigate the environmental footprint of road infrastructure [5]. In this regard, the use of reclaimed asphalt pavement (RAP) and reclaimed aggregate (RA) plays a major role in reducing virgin material consumption, lowering transportation costs, and enhancing economic efficiency [6,7].

### 1.2. Cold In-Place Recycling with Foamed Bitumen

Cold in-place recycling with foamed bitumen (CIR-FB) has gained increasing relevance in municipal road rehabilitation due to its ability to reuse up to 100% of reclaimed materials while ensuring adequate structural performance [8]. The absence of heating significantly reduces energy consumption and emissions [9], and CIR-FB has been widely validated in international practice for low- and medium-volume roads [10,11]. A significant advantage of CIR-FB is the reduced curing period required before the treated base layer can be opened to traffic [12].

Comparable research initiatives in North America and Asia also demonstrate that cold recycling with foamed bitumen can ensure durability and cost-effectiveness in low-volume roads, reinforcing the global applicability of such methods. Studies conducted by the Federal Highway Administration (FHWA) in the United States have confirmed that pavements using cold in-place recycling (CIR) with foamed bitumen can achieve performance levels equivalent to those with conventional hot-mix asphalt for secondary and rural roads, while reducing construction costs by 20–40% and cutting GHG emissions by up to 50% [10,13]. Similar outcomes were reported in Virginia, where the adoption of CIR-based methods significantly decreased life-cycle environmental impacts [14]. Research in Australia and Canada demonstrated that foamed bitumen-stabilized materials achieve high stiffness and fatigue performance under accelerated loading, ensuring long-term structural reliability in diverse climatic conditions [11,15]. Asian case studies, particularly from China and India, also confirm that cold recycling with foamed bitumen improves rutting resistance and reduces energy consumption compared to hot-mix technologies [16]. These findings collectively confirm that foamed bitumen-based cold recycling is a globally recognized, sustainable, and technically reliable approach to municipal road rehabilitation.

CIR-FB technique involves milling of existing asphalt layers, mixing them in situ with foamed bitumen, Portland cement and water, followed by re-compacting the mixture to form a new bound base. The temporary reduction in bitumen viscosity during foaming ensures good coating and adhesion of RAP, resulting in durable and sustainable pavement layers [12,17]. Similar applications of foamed bitumen for soil and subbase stabilization have also been documented [18]. The cost and eco-effectiveness of cold in-place recycled mixtures with foamed bitumen have been evidenced in recent studies [19], supporting the potential of the CIR-FB technique under variable subgrade conditions. One of key advantages of the recycling technology using foamed bitumen is the ability to use inferior quality mineral aggregate if needed and the tendency of foamed bitumen to bond effectively to fine aggregate.

### 1.3. Environmental and Mechanical Assessment Framework

Environmental and economic evaluations, particularly life cycle assessment (LCA) and Life cycle cost assessment (LCCA), demonstrate that cold recycling and WMA can reduce the carbon footprint of pavement projects by 30–50% compared to conventional methods [9,20]. Comparative LCA studies of porous asphalt with reclaimed aggregate and alternative binders further highlight the sustainability benefits of recycling-based solutions [21,22,23]. Additionally, studies have shown that incorporating up to 50% RAP can provide both mechanical and environmental advantages without compromising performance [24]. From an economic standpoint, RAP and secondary materials can reduce virgin aggregate demand by 20–60%, depending on the reconstruction method and local conditions [9,25].

In summary, it is well established that pavement rehabilitation methods utilizing reclaimed materials not only meet structural and durability requirements but also significantly reduce environmental burdens and project costs. Integrating environmental analysis results into design, supported by case studies and SWOT (strengths, weaknesses, opportunities and threats) analysis, provides a robust framework for developing optimal road rehabilitation strategies aligned with sustainable development goals.

As for the pavement design, mechanistic–empirical methods provide more accurate long-term performance predictions than purely empirical approaches, particularly when recycled materials are incorporated. Ibrahim et al. [26] integrated the Mechanistic–Empirical Pavement Design Guide analysis into an LCA framework and demonstrated that incorporating use-phase mechanistic–empirical results can shift environmental rankings and support low-carbon material selection in pavement rehabilitation.

### 1.4. Scope, Objectives and Novelty of the Study

In Poland, municipal roads—mainly access and local routes—constitute nearly 250,000 km, accounting for approximately 59% of the national public network. These pavements are most prone to premature degradation due to frequent service loading and limited maintenance budgets, and traditional repairs often fail to deliver long-term structural improvement [27]. To address this challenge, a representative municipal road section was selected as a test case to evaluate whether reconstruction based on reclaimed materials can outperform a conventional virgin-material solution in terms of performance, resource use and environmental load.

Although CIR-FB technologies have been widely studied, the existing literature rarely provides an integrated assessment combining mechanistic–empirical structural performance predictions with LCA, LCCA and material flow evaluation. Moreover, evidence for municipal roads in Central and Eastern Europe remains limited, despite climatic conditions, RAP characteristics and budget constraints differing markedly from regions most frequently examined. These gaps indicate a need for holistic, region-specific evaluations that quantify the engineering performance as well as the environmental and economic implications of CIR-FB reconstruction compared with conventional rehabilitation approaches.

The study compares two pavement designs: a reference structure constructed from virgin aggregates and new asphalt layers, and a recycling-based alternative incorporating CIR-FB using RAP from the wearing and binding layer and RA from the existing unbound aggregate base. The assessment integrates laboratory stiffness testing, mechanistic–empirical performance analysis, material flow quantification, LCA, LCCA and SWOT interpretation, enabling a full evaluation across structural, environmental and economic domains.

The central hypothesis is that properly characterized and structurally integrated reclaimed materials can increase pavement fatigue life while simultaneously reducing material extraction, emissions and cost. The analysis therefore focuses on parameters most relevant for road authorities: predicted fatigue life, virgin resource demand, demolition volume, transport intensity and overall carbon footprint. A complementary SWOT assessment [28,29] extends these quantitative findings by highlighting implementation conditions, risks and decision constraints relevant to practice.

The developed methodology is transferable to other Central and Eastern European regions where similar climatic stresses and budgetary limitations shape road rehabilitation. The terminology used in the present paper is consistent with the EU waste management hierarchy [30,31], where reclaimed refers to direct reuse of RAP and RA, while recycled denotes reprocessed mixtures such as CIR-FB base layers.

## 2. Materials and Methods

### 2.1. Characteristics of the Analyzed Road Section

The analysis covered a deteriorated 1 km long two-lane, single carriageway section of a municipal road with a crowned cross section (2.75 m wide lanes), located in the southeastern part of Poland, outside the built-up area.

**Figure 1:**
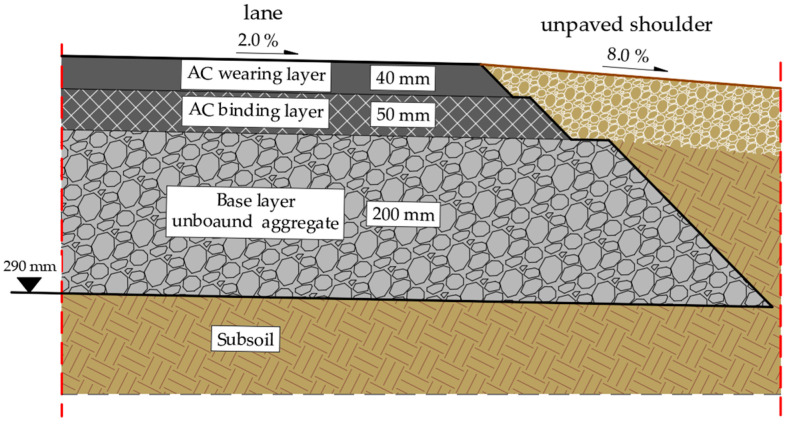
Existing pavement structure of the analyzed road section.

The structural layers of the road pavement in the existing state (Figure 1) were made of the following materials:

- Asphalt concrete (AC) wearing layer with a maximum aggregate size of 11 mm and a thickness of 40 mm;
- Asphalt concrete (AC) binding layer with a maximum aggregate size of 16 mm and a thickness of 50 mm;
- Base layer made of an unbound aggregate mixture graded 0/31.5 mm (i.e., containing particles from 0 mm up to a maximum size of 31.5 mm), mechanically stabilized, with a thickness of 200 mm.

The pavement structure of the existing road section was constructed on a subsoil made of permeable soils (medium sands) with the California bearing ratio (CBR) = 20%, determined in accordance with the EN 13286-47 standard [32]. Water conditions have been defined as good, in which the highest level of free groundwater table occurs at a depth > 2 m below the underside of the pavement structure. At this depth, moisture does not affect subsoil performance, and therefore, no additional drainage or subgrade improvement measures are required.

In this study, the term ‘road surface structure’ refers to the upper pavement system, including the asphalt wearing course, the binder course, and the asphalt base layer.

The average daily traffic volume for the examined road section is 3550 vehicles/day. For the analysis of pavement structure, the prospective average daily traffic was used as a representative traffic load, considering only heavy vehicles (0.3% share in daily traffic). In this municipal road, heavy-vehicle traffic consists almost exclusively of buses (12 per day, assuming 1% annual growth rate due to the stable traffic pattern and local functional role of the road). The ESAL value was calculated using the load equivalency relationship expressed by Formula (1).

(1) $${ESAL}_{100\;kN}=f_{1}\times f_{2}\times f_{3}\times (N_{C}\times r_{C}+N_{C+P}\times r_{C+P}+N_{A}\times r_{A})$$

where ESAL_100kN_ is the cumulative number of equivalent 100 kN single axle loads; $N_{C}$ , $N_{C+P}$ and $N_{A}$ are the 20-year numbers of trucks without trailers, trucks with trailers, and buses, respectively; $r_{C}$ , $r_{C+P}$ , $r_{A}$ —are the corresponding axle-load equivalency factors; and $f_{1}$ , $f_{2}$ , $f_{3}$ denote the lane-distribution, lane-width, and grade-slope factors.

The adopted equivalency factor for buses was $r_{A}$ = 1.05, and the coefficients $f_{1}$ = 1.0, $f_{2}$ = 1.13, and $f_{3}$ = 1.0 were taken from the national pavement design catalog [33]. On the basis of the analysis and calculation of the traffic forecast (20 years) in accordance with the catalog [33], the number of ESAL_100kN_ = 0.1 × 10^6^ per lane was determined, which corresponds to the traffic load category 0.09 × 10^6^ < ESAL_100kN_ ≤ 0.50 × 10^6^, i.e., a light traffic load. In international systems, this corresponds to the least intensive traffic categories, e.g., LT (low-traffic volume for ESAL ≤ 1 mln) in the guidelines [3].

The traffic analysis demonstrated that the existing pavement structure (Figure 1) did not comply with the requirements in terms of traffic load. The thickness and type of layers of the structure corresponded to the lowest traffic category (ESAL 100 kN < 0.09 × 10^6^), which indicates the need for increased load-bearing capacity and to adapt it to current traffic needs. In order to determine the scope of the renovation/reconstruction, an assessment of the technical condition of the pavement was carried out.

The basis for determining the technical condition of the visual-distress evaluation criteria is a non-automated pavement-condition survey. Indicators of pavement crack and surface condition were determined in accordance with the guidelines [4].

When determining the scope of pavement repairs, the following criteria were taken into account in accordance with the national recommendations [34]:

- Partial repair, if no more than 20% of the surface is damaged;
- Total repair, if more than 20% of the surface is damaged.

A visual assessment of the pavement condition identified numerous surface defects, including transverse and longitudinal cracks, patches, raveling and isolated potholes, indicating advanced deterioration of the existing structure. Table 1 summarizes the occurrence of the main pavement damage types identified on the surveyed section.

In accordance with the Polish pavement condition assessment system [35], the road section was classified as condition class C (warning level), requiring comprehensive repair. The determined area of damage (20.1%) slightly exceeds the threshold value of 20% specified in the catalog [34], which formally qualifies the analyzed road section for full-depth reconstruction. Detailed photographic documentation, full distributions of damage types and their spatial variability are provided in Appendix A.

### 2.2. Materials and Technologies Considered in the Study

#### 2.2.1. Pavement Reconstruction Technologies Considered

Two alternative reconstruction approaches were analyzed for the municipal road section:

- Traditional reconstruction, involving the complete removal of the existing pavement layers and the use of only virgin materials to build a structure with increased bearing capacity;
- Recycling-based reconstruction, utilizing CIR-FB and relying on the reclaiming and reuse of materials obtained from the existing asphalt layers (RAP) and the existing unbound base layer (RA).

#### 2.2.2. Materials Used in the Design Variants

The following materials were considered in the structural design of the two reconstruction variants:

- Virgin materials:
  ○ Asphalt concrete (AC), compliant with the requirements of EN 13108-1 [36] standard;
  ○ Unbound aggregate mixture, compliant with the requirements of the EN 13285 [37], EN 13242+A1 [38] standards;
  ○ Foamed bitumen, compliant with the requirements of the EN 12591 [39] as the primary binder in the CIR-FB mixture;
  ○ Cement with properties in accordance with EN 197-1 [40] as an active filler.
- Reclaimed materials:
  ○ Reclaimed asphalt pavement (RAP), obtained from the existing wearing and binding layers and tested according to EN 13108-8 standard [41];
  ○ Reclaimed aggregate (RA), obtained from the existing unbound base layer and evaluated in accordance with EN 13242+A1 standard [38].
- Recycled mixture:
  ○ Cold in-place recycled mixture with foamed bitumen (CIR-FB), produced from RAP and RA with the addition of foamed bitumen (FB) and cement (C) as binders.

#### 2.2.3. Laboratory Testing of Reclaimed Materials (RAP and RA)

To assess the suitability of reclaimed materials for reuse, laboratory tests were conducted on reclaimed asphalt pavement from the wearing layer (RAP-1), the binding layer (RAP-2), and reclaimed aggregate (RA) obtained from the existing unbound base layer.

The laboratory evaluation of RAP included the following:

- Particle size distribution—determined in accordance with EN 933-1 [42];
- Binder content—measured by solvent extraction using tetrachloroethylene (EN 12697-1 [43]);
- Recovered binder properties, including penetration and softening point—determined according to EN 12697-3+A1 [44], EN 1426 [45] and EN 1427 [46];
- Classification of reclaimed asphalt material based on maximum particle size and aggregate grading—according to EN 13108-8 [41];
- Visual inspection of aggregate lithology.

The RA material was evaluated to determine its suitability for reuse in the CIR-FB mixture and in unbound layers. The assessment included the following:

- Particle size distribution—determined in accordance with EN 933-1 [42];
- Geometric properties—evaluated in accordance with EN 13242+A1 [38];
- Physical properties—assessed in accordance with EN 13242+A1 [38], as required for aggregate used in unbound and hydraulically bound layers;
- Visual inspection—to identify aggregate lithology and assess material uniformity.

#### 2.2.4. CIR-FB Mixture Design and Testing Methods

The CIR-FB mixture was designed following a laboratory procedure consistent with national guidelines and European standards, with particular focus on determining the optimum content of foamed bitumen (FB), cement (C), and water required to ensure adequate strength, stiffness, and moisture resistance.

The mixture design included the following steps:

- Assessment of combined mineral gradation of RAP and RA and verification against recommended CIR gradation curves (EN 933-1 [42]);
- The selection of FB and C contents was carried out by preparing a matrix of trial mixtures with the following binder combinations:
  ○ C = 1.5% with FB = 2.0% or 2.5%;
  ○ C = 2.0% with FB = 2.5% or 3.0%.
- Determination of optimum moisture content OMC of the mineral-cement mix based on compaction to maximum dry density using the modified Proctor procedure (EN 13286-2 [47]);
- Preparation and compaction of specimens for mechanical testing at OMC.

The final CIR-FB mixture composition (gradation, FB and C contents and OMC) was established based on the laboratory optimization procedure.

The following mixture properties were evaluated in accordance with the applicable EN standards:

- Indirect tensile strength (*ITS*) specifications in dry (*ITS_d_*) and wet (*ITS_w_*) conditions acc. EN 12697-23 [48] standard are as follows:
  ○ Cylindrical specimens with a diameter of 101 mm;
  ○ Test temperature: 25 °C;
  ○ Specimens conditioned for 24 h at 25 °C before testing.
  ○ Wet specimens conditioned by water soaking for 24 h at 25 °C before testing.
- Tensile strength ratio (moisture susceptibility)**,** expressed as *TSR* = *ITS_w_*/*ITS_d_* acc. [49];
- Bulk density ρ_b_ acc. EN 12697-5 [50] standard (water displacement) and EN 12697-6 [51] standard (geometric method);
- Air void content *V*_a_, acc. EN 12697-8 [52] standard;
- Unconfined compressive strength (*UCS*)—The uniaxial compressive strength at 25 °C, acc. EN 13286-41 [53] standard, is as follows:
  ○ Specimens compacted to OMC with the use of the Proctor method acc. EN 13286-50 [54] standard;
  ○ Conditioning: 28 days of air-dry curing at 20 ± 5 °C and 95% RH (standard CIR-FB curing regime).
- Stiffness modulus under indirect cyclic loading at 25 °C acc. EN 12697-26 [55] standard—*IT-CY* procedure is commonly adopted in cold-recycling studies:
  ○ Test temperature: 25 °C;
  ○ Loading: Loading time: 125 ms, pulse repetition period: 3 s;
  ○ Conditioning: 24 h at 25 °C prior to testing.

Test specimens were compacted using a Marshall impact compactor with 75 blows per side, in accordance with to EN 12697-30 [56].

#### 2.2.5. Comparative Assessment Methods for the Design Variants

The designed variants of the road pavement reconstruction were compared in terms of engineering performance, economic and ecological aspects in terms of the following indicators:

- Pavement fatigue life (ESAL 100 kN);
- Mass of demolition waste and volume of material requiring disposal (Mg);
- Demand for new virgin materials (Mg);
- Number of heavy goods vehicle (HGV) trips associated with material delivery and removal (trips);
- Transport work associated with the delivery and export of materials (t-km and veh-km);
- GHG emissions associated with material production, transport and construction activities (kg CO_2_-eq);
- Total construction-phase life-cycle costs, including material, transport and site-operation costs (EUR).

The transport work associated with the delivery of construction materials—including natural aggregate, cement, bituminous binders, asphalt mixtures, and reclaimed demolition materials—was expressed in ton–kilometer (t-km) and vehicle–kilometers (veh-km). The t-km indicator represents the product of the transported mass (in Mg) and the distance traveled (in kilometers) and serves as the standard measure of freight transport performance, as defined by Eurostat and OECD transport statistics [57,58]. The veh-km unit describes the total distance traveled by vehicles, including both loaded trips and unloaded return trips, and provides a consistent basis for assessing the environmental and logistical efficiency of material transport in road construction and maintenance projects.

A construction-phase LCA was performed using an inventory-based calculation approach. Total emissions were obtained as the sum of the following:

- A1–A3 material production (mass × emission factor);
- A4 transport emissions (t-km × emission factor);
- A5 construction and demolition operations.

The GHG emissions were calculated by multiplying the mass of each material by its corresponding CO_2_ emission factor, and by adding emissions associated with transport and construction-stage activities. Emission factors for construction materials were sourced from the ecoinvent v3.12 database [59] and the ICE Database v4.1 [60]—widely recognized datasets used in infrastructure-related LCA and consistent with ISO 14040/14044 [61,62]. Transport-related emissions were calculated per ton–kilometer using average European emission intensities for heavy-duty vehicles.

A construction-phase LCCA was carried out analogously by multiplying inventory quantities by unit-price coefficients. Total cost was calculated as C1 + C2 + C3, where C1 covers material procurement, C2 transport and haulage, and C3 construction and site operations. Since only the reconstruction stage was evaluated, discounting was not applied.

### 2.3. Method of Designing the Road Surface Structure

To compare the designed pavement structures, an empirical–mechanistic method was applied, combining classical mechanics with empirical data and performance-based models. This approach enables a reliable prediction of pavement durability under varying traffic, climatic, and subgrade conditions. In Polish engineering practice, pavement design is based primarily on the fatigue criterion developed by the Asphalt Institute and included in the Mechanistic–Empirical Pavement Design Guide [63], as well as on the recommendations of the National Catalogue of Typical Flexible and Semi-Rigid Pavement Structures [33]. In recent years, the implementation of AASHTO mechanistic–empirical transfer functions has further aligned the Polish design methodology with international standards [64].

The design process considers the critical tensile strain at the bottom of the bituminous layer (*ε_H_*) and the vertical compressive strain at the top of the subgrade (*ε_V_*). These parameters are related to fatigue cracking and permanent deformation, respectively. The general fatigue criterion adopted in the design is expressed as follows (2)–(4) [65]:

(2) $$N_{f}=7.3557\cdot 10^{-6}\cdot C\cdot {k^{\prime }}_{1}\cdot \varepsilon_{H}^{-3.9492}\cdot {\left|E^{*}\right|}^{-1.281}$$

(3) $$C=10^{M}$$

(4) $$M=4.84\cdot {[V}_{b}/(V_{b}+V_{a})-0.69]$$

where $N_{f}$ —fatigue cracking life (with 20% of wheel tracks cracked), expressed in ESAL 100 kN, $\varepsilon_{H}$ —horizontal tensile strains at the bottom of the bituminous layers (µε), |E*|—dynamic stiffness modulus of the bituminous layer (MPa), ${k^{\prime }}_{1}$ —parameter dependent on the thickness of bituminous layers, $V_{b}$ —volumetric effective binder content (%), $V_{a}$ —air void content (%).

The calculated number of load repetitions is associated with damage D = 1 for the bituminous layer. By calculating the actual fatigue damage using the Miner rule, it is possible to predict the bottom-up cracking in percent of the total lane area through the transfer function (5).

(5) $$FC_{bottom}=100/(1+\exp(-2\cdot {C^{\prime }}_{2}+{C^{\prime }}_{2}\cdot {log}_{10}(D\cdot 100)))$$

where ${C^{\prime }}_{2}$ = −2.40874 − 39.748·(1 + h_ac_/2.54)^−2.856^ and h_ac_—thickness of bituminous layers (cm).

In this study, the empirical–mechanistic method was applied to both the conventional solution (V_1_-N), based on virgin materials, and the recycling-based variant (V_2_-Rc), using RAP and RA. For each case, fatigue life was determined according to the Polish adaptation of MEPDG criteria, considering the stiffness modulus and volumetric composition of asphalt and recycled mixtures, and applying the Miner cumulative damage rule.

## 3. Results

### 3.1. Laboratory Evaluation of Reclaimed Pavement Materials

One of the methods of strengthening the pavement structure analyzed in this study involves the use of materials reclaimed from the existing structural layers. RAP is a valuable secondary material increasingly incorporated into the production of new asphalt mixtures used in pavement rehabilitation and reconstruction. Together with RA, it can also be used in base layers constructed using CIR technology. However, the use of lower-quality reclaimed materials carries the risk of producing a structural layer with insufficient mechanical performance. To demonstrate the suitability of the waste materials from the analyzed road section for reuse, basic laboratory tests of their properties were performed.

**Figure 2:**
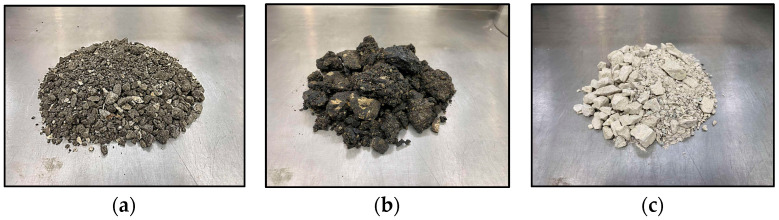
Reclaimed materials obtained from the existing pavement layers: (**a**) RAP-1—wearing layer milling; (**b**) RAP-2—binding layer milling; (**c**) RA—unbound aggregate base.

Reclaimed materials obtained from the following layers of the existing structure were evaluated:

- Reclaimed asphalt pavement from the wearing layer (RAP-1) and the binding layer (RAP-2);
- Reclaimed aggregate (RA) from the base layer, shown in Figure 2.

**Figure 3:**
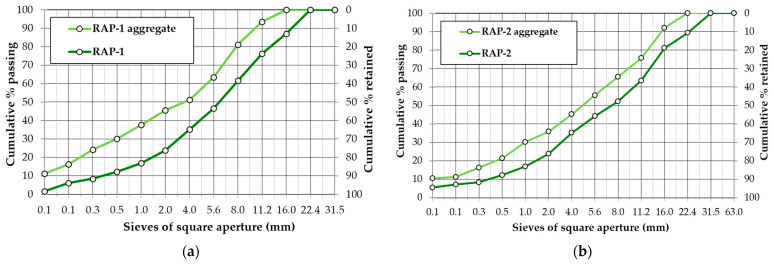
Particle size distribution of RAP and RAP aggregate (after bitumen extraction): (**a**) RAP-1; (**b**) RAP-2.

The particle size distribution of the reclaimed asphalt pavement and the aggregate reclaimed from it (after solvent extraction of the binder) was determined according to EN 933-1 [42] and is shown in Figure 3a (wearing layer material) and Figure 3b (binding layer material).

The solvent extraction showed that the binder content in asphalt mixtures was 5.8% for the material reclaimed from the wearing layer and 4.6% for the binding layer. The grain-size distribution of the aggregate reclaimed from RAP, together with its binder content, meets the requirements of the national standard [66] for asphalt mixtures used in pavements designed for light traffic categories.

Based on the maximum particle size of reclaimed asphalt mixture (U) and the aggregate grain size after binder extraction of the RAP-1 material (in accordance with the 13108-8 standard [41]), it was designated as 16 RA 0/11.2, that is, reclaimed asphalt material with aggregate size of 11.2 mm and asphalt particles of a maximum size of 16 mm. RAP-2 was designated as 22 RA 0/16, i.e., reclaimed asphalt material with aggregate size of 16 mm and asphalt particles of a maximum size of 22.4 mm. Based on visual evaluation, it was found that the mineral mixture extracted from RAP-1 consists of gabbro and limestone, as well as RAP-2 of limestone aggregate.

Basic tests were carried out according to the standard [41] for the bitumen extracted from the RAP material. The results of the analysis are presented in Table 2.

Based on the basic tests of the bitumen reclaimed from RAP, it can be concluded that although the binders in the RAP-1 and RAP-2 are aged due to their fatigue life, they fulfill the requirements for use in recycled asphalt mixtures [66].

**Figure 4:**
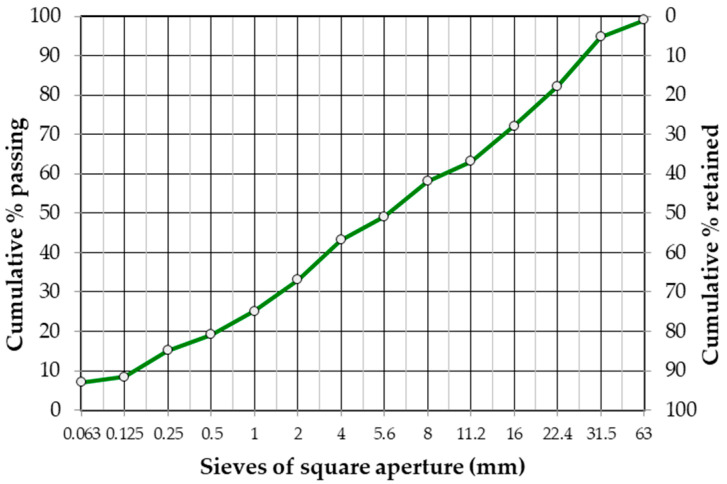
Particle size distribution of RA from the existing base layer.

From the existing surface of the analyzed road section, it is possible to extract the natural aggregate reclaimed from the base layer. The use of this type of material to build a new pavement structure or rebuild an existing one would require additional crushing, screening, and grading. In the case of cold recycling, it is possible to use this material on-site without additional processing. The results of the tests to determine the particle size distribution of the RA according to EN 933-1 [42] are shown in Figure 4. The geometric and physical parameters of the RA were determined and listed in Table 3.

The aggregate reclaimed from the existing base layer is characterized by a grain size of 0/31.5 mm with a sieving curve (Figure 4), as well as physical and geometric properties (Table 3) that meet the requirements of the EN 13242+A1 standard [38] and national requirements [67]. Based on visual inspection, the reclaimed mineral mixture was preliminarily identified as consisting of limestone aggregate. This is plausible because limestone is the predominant aggregate type produced in the local quarries.

The bitumen and aggregate reclaimed from RAP exhibit properties comparable to those of virgin materials typically used in asphalt mixtures for structural layers designed for light-traffic pavements. This confirms the feasibility of using RAP from the existing wearing course in the production of new asphalt binding layers, while RAP obtained from the binder course may be incorporated into the CIR-FB mixture.

**Table 3:** Geometric and physical properties of RA from existing base layer acc. EN 13242+A1 [39].

Property	Test Method	Result	Category
Grading	EN 933-1 [42]	Grading curve (Figure 4)	G_A_85
Percentage of grains with crushed or broken surfaces and fully rounded grains (%)	EN 13242+A1 [38]	99/0	C_90/3_
Particle density, ρ_a_ (Mg/m^3^)	EN 1097-6 [68]	2.720	-
Flakiness index (%)	EN 933-3 [69]	19	FI_20_
Shape index (%)	EN 933-4 [70]	24	SI_40_
Fines content (%)	EN 933-1 [42]	3.5	f_4_
Resistance to fragmentation, Los Angeles test method, 10/14 mm (%)	EN 1097-2 [71]	32	LA_35_
Freeze–thaw resistance (%)	EN 1367-1 [72]	1.2	F_2_

RA material from the existing base layer demonstrates physical and mechanical properties comparable to continuously graded aggregates produced in quarries. Therefore, it may be effectively reused as a component in CIR-FB base layers.

The results confirm that the recycling variant (V_2_-Rc) can incorporate both RAP and RA from the existing pavement structure, reducing the demand for virgin resources. However, ensuring long-term performance requires appropriate material quality control. As emphasized by Tarsi et al. [73], variability in RAP properties (gradation, binder aging, mineral composition) may affect mixture homogeneity and fatigue resistance if not properly characterized.

Accordingly, a thorough laboratory evaluation is required prior to both RAP incorporation into new asphalt binding layer and RA/RAP utilization within CIR-FB mixtures.

### 3.2. CIR-FB Mixture Design and Laboratory Performance Evaluation

Based on the laboratory characterization of reclaimed materials (Section 3.1), four foamed-bitumen recycled mixtures were produced using RAP-2 and RA with different cement/foamed bitumen contents (C/FB = 1.5/2.0%, 1.5/2.5%, 2.0/2.5% and 2.0/3.0%).

The bituminous binder used in the investigated mixtures was a paving-grade bitumen 50/70, with penetration between 50 × 0.1 mm and 70 × 0.1 mm at 25 °C, compliant with the requirements of EN 12591 [39] standard. Foamed bitumen was produced from this base binder, and its foaming characteristics were optimized prior to CIR-FB mixture preparation. The optimum foaming parameters were obtained as follows:

- Foaming water content: 2%,
- Maximum expansion ratio: 12.5;
- Foam half-life: 9.0 s.

The hydraulic binder used in the CIR-FB mixtures was Portland-composite cement CEM II/B-V 42.5 R, compliant with EN 197-1 [40], containing siliceous fly ash as a main mineral addition and characterized by high early strength development (class R), which is favorable for cold-recycled mixtures requiring accelerated stiffness gain.

**Figure 5:**
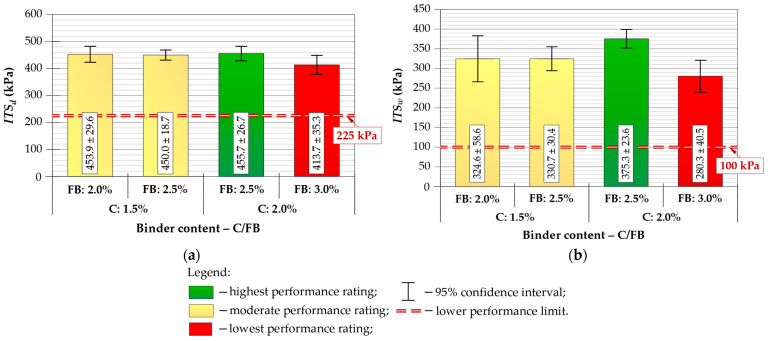
Indirect tensile strength of CIR-FB specimens with marked performance rating with lower acceptance limits acc. [74]: (**a**) *ITS_d_* (**b**) *ITS_w_*.

**Figure 6:**
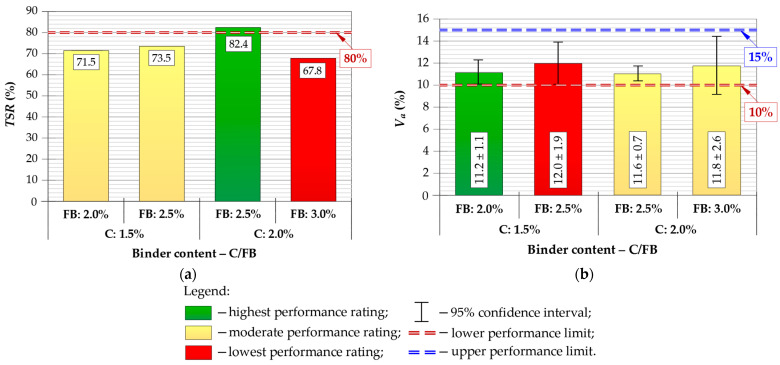
Moisture susceptibility and air void content of CIR-FB specimens with marked performance rating: (**a**) *TSR* with lower acceptance limits acc. [49]; (**b**) *V_a_* with lower/upper acceptance limits acc. [74].

**Figure 7:**
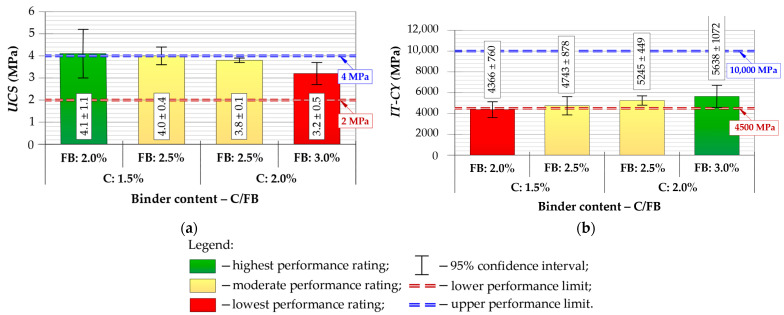
Unconfined compressive strength and stiffness modulus of CIR-FB specimens with marked performance rating: (**a**) *UCS* with lower/upper acceptance limits acc. [49]; (**b**) *IT-CY* with lower/upper acceptance limits acc. [75].

Six specimens were prepared for each composition, and mean values with 95% confidence intervals were calculated for all performance indicators. The results of *ITS*, *TSR*, *V_a_*, *UCS* and *IT-CY* stiffness modulus tests are presented in Figure 5, Figure 6 and Figure 7, with performance ratings and acceptance limits.

All CIR-FB mixtures satisfied the minimum required *ITS_d_* and *ITS_w_* parameters criteria according to [74]. As shown in Figure 5, the C/FB = 2.0/2.5% mixture exhibited the highest tensile performance, reaching *ITS_d_* ≈ 456 kPa and *ITS_w_* ≈ 375 kPa. Moisture-related strength reduction was also the smallest in this composition, resulting in *TSR* ≈ 82%, which is the only mixture meeting the recommended threshold of *TSR* ≥ 80% (Figure 6a), appropriate for Central-Eastern European climatic conditions [75] and compliant with recommended lower limit [49]. *TSR* parameters for the remaining binder configurations ranged from approximately 68–74%, confirming insufficient moisture resistance for long-term structural use, particularly under the local climate conditions.

Air void contents for all mixtures remained within acceptable limits (Figure 6b). The lowest *V_a_* was recorded for C/FB = 1.5/2.0%, while 2.0/2.5% and 2.0.3.0% mixtures produced an intermediate value compliant with specification limits. The highest *V_a_* occurred in the 1.5/2.5% mixture, though still within the permissible range. These results confirm that all tested mixtures achieved adequate compaction and internal structure, while the 2.0/2.5% variant offered a more preferable balance of compacted density and mechanical response.

For the *IT-CY* assessment, lower and upper stiffness boundaries were adopted in accordance with national requirements [75], reflecting criteria commonly used by road authorities operating under similar Central-Eastern European climatic conditions. The stiffness modulus test (*IT-CY*) showed an opposite trend to *UCS* (Figure 7). The mixture with 1.5/2.0% C/FB exhibited insufficient stiffness (below the required 4500 MPa), whereas the highest modulus (≈5638 MPa) was obtained for 2.0/3.0%. The 2.0/2.5% mixture reached an intermediate value, meeting the specification threshold and ensuring a balanced structural response. The resulting *IT-CY* modulus values will be used later in fatigue-life calculations for pavement design.

Based on the laboratory performance evaluation, the C/FB = 2.0/2.5% mixture was selected as the final composition for use in further structural, economic and environmental analysis. Although the 2.0/3.0% mixture achieved the highest *IT-CY* parameter, its higher rigidity may increase the risk of reflective cracking and reduce fatigue tolerance under long-term traffic loading—an undesirable effect for municipal-class pavements.

The 2.0/2.5% C/FB mixture demonstrated the most favorable overall balance of properties:

- *ITS_d_* and *ITS_w_*: high and stable tensile performance under both dry and wet conditions;
- *TSR* ≥ 80%: the only acceptable composition;
- *UCS* ≈ 3.8 MPa: compliant mid-range value, within the required 2–4 MPa range, ensuring sufficient compressive capacity without excessive brittleness;
- *IT-CY* within 4500–10,000 MPa: structurally adequate modulus, unlike 1.5/2.0% which fell below the lower threshold;
- *V_a_* within specification limits 10–15%: confirming appropriate compaction and internal structure.

Considering the above criteria, C/FB = 2.0/2.5% was identified as the optimum CIR-FB mixture, offering the best balance between strength, durability, moisture resistance and stiffness. This composition was therefore selected as the final CIR-FB mixture for further fatigue, structure and LCAs/LCCAs.

The final mixture was designed with a total asphalt binder content of 4.1%. The asphalt binder contained in the RAP contributed 1.6% to the CIR-FB mixture, while the added foamed bitumen contributed 2.5%. The total binder content complied with the relevant recommendations (not exceeding 6%) [75].

The optimum moisture content (OMC) determined for the final CIR-FB mixture was 5.6% based on compaction to maximum dry density using the modified Proctor procedure EN 13286-2 [47].

The bulk density of the CIR-FB mixture determined according to EN 12697-5 [50] was 2.150 Mg/m^3^, while the maximum density determined according to EN 12697-6 [51] was 2.392 Mg/m^3^.

**Figure 8:**
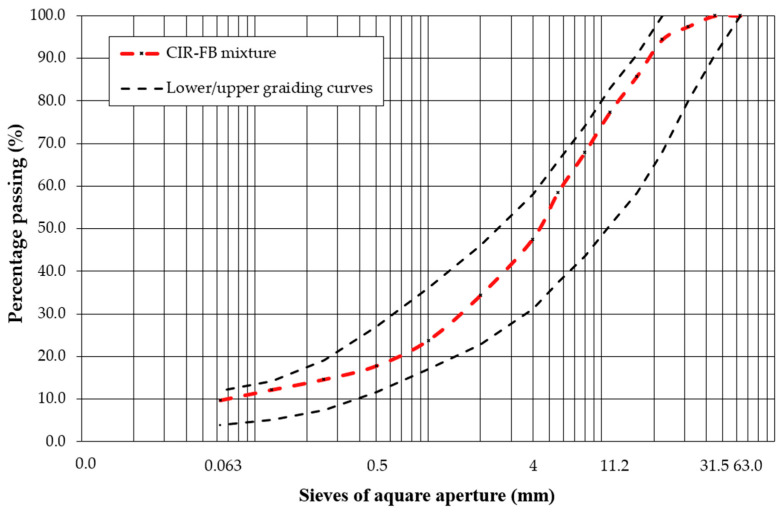
Particle size distribution of the CIR-FB mixture with boundary curves according to the recommendations [74].

The composition of the investigated final mixture is shown in Table 4, while its designed particle size distribution is shown in Figure 8.

No additional mixing water was introduced into the formulation—the optimum moisture content equaled the natural moisture level of the recycled constituents, including the water contained in the foamed bitumen.

The gradation curve of the final mixture (Figure 8) lies fully within the recommended CIR-FB boundary curves [75], confirming suitability for recycling-based base course construction.

### 3.3. Pavement Reconstruction and Fatigue Life Analysis

To estimate the environmental and financial benefits of the designed structures, typical and alternative solutions for complete reconstruction of the pavement structure (in line with the national technical document [35]) were selected for comparison purposes, but with equal thicknesses. Two variants of material and technological solutions for the reconstruction of the existing pavement structure were proposed, resulting from the extent of the damage, the soil and water conditions and the traffic load. The chosen design approach follows directly from the pavement condition evaluation, where 20.1% surface damage (class C—warning level) justified full-depth rebuilding, rather than local treatment (Appendix A). In the V_1_-N variant, the entire existing structure was removed because this option was intentionally defined as a fully conventional reconstruction scenario relying solely on virgin materials. Therefore, retaining 100 mm of the existing base—although technically possible—was not included, to ensure conceptual consistency and preserve a clear contrast with the recycling-based V_2_-Rc variant.

The following design assumptions were taken into account:

- Minimum design traffic load ESAL 100 kN > 0.50 × 10^6^;
- Good water conditions (the highest level of free groundwater table occurs at a depth below the bottom of the pavement structure > 2 m);
- Native soils in the subsoil, permeable, with CBR ≥ 10%;
- Raising the existing longitudinal profile of the pavement by 80 mm.

Variant 1 (V_1_-N) of the reconstruction of the pavement structure takes the following into account:

- Complete demolition of the existing road surface structure (dismantling of asphalt and aggregate layers) along with the removal and disposal of materials;
- Compaction and profiling of the prepared roadbed made for the new pavement structure;
- Construction of new layers using virgin materials.

**Figure 9:**
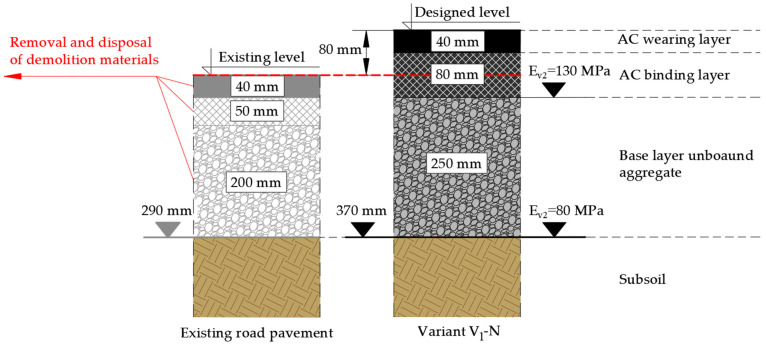
Existing and designed pavement layer layout based on virgin materials for variant V_1_-N (E_v2_—required secondary elasticity modulus).

In the V_1_-N variant, the following structural layers of the road surface (Figure 9) with an unbound aggregate layer were adopted, among other things, due to the close location (approx. 5 km) of a mineral quarry were designed, on the basis of virgin materials and with a total thickness of 370 mm:

- Asphalt concrete (AC) wearing layer with maximum aggregate size of 11 mm, thickness 40 mm;
- Asphalt concrete (AC) binding layer with maximum aggregate size of 16 mm, thickness 80 mm;
- Base layer made of an unbound aggregate C_NR_ with a grain size of 0/31.5 mm, mechanically stabilized, thickness 250 mm.

Variant 2 (V_2_-Rc) of the reconstruction of the pavement structure takes into account the maximum use of materials reclaimed from the worn structural layers:

- Milling of the existing pavement wearing layer and its transport to the asphalt plant to enable the use of the RAP for the binding layer;
- Paving of the base layer made of in situ produced CIR-FB using materials from the existing asphalt concrete binding layer and aggregate from half of the thickness of the existing unbound base layer;
- Paving a binder layer containing 20% RAP obtained from the existing wearing layer.

The variant V_2_-Rc assumes 92% of the use of existing structural layers in recycling technology for the construction of the binding layer and the base layer.

**Figure 10:**
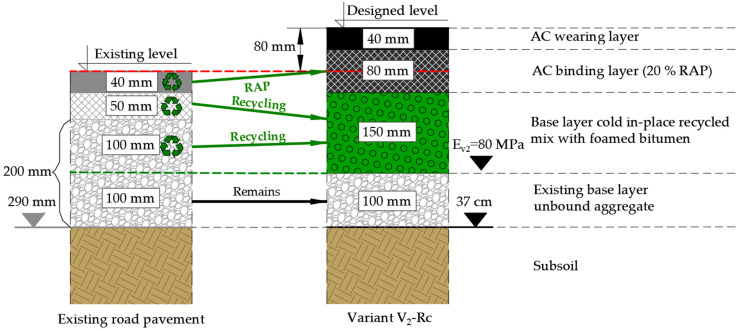
Existing and designed pavement layer layout based on reclaimed materials for variant V_2_-Rc (E_v2_—required secondary elasticity modulus).

In the V_2_-Rc variant, the following structural layers of the road surface (Figure 10) were designed using reclaimed materials with a total thickness of 370 mm:

- Asphalt concrete (AC) wearing layer with a maximum aggregate size of 11 mm and a thickness of 40 mm;
- Asphalt concrete (AC) binding layer with a maximum aggregate size of 16 mm and the use of a 20% RAP with a thickness of 80 mm;
- Base layer made of a cold in-place recycled mixture with foamed bitumen (CIR-FB), with the use of RAP from the milled binding layer and RA from half the thickness (100 mm) of the existing unbound aggregate base layer, with a thickness of 150 mm;
- Existing base layer made of an unbound mixture of aggregate, with a thickness of 100 mm.

**Figure 11:**
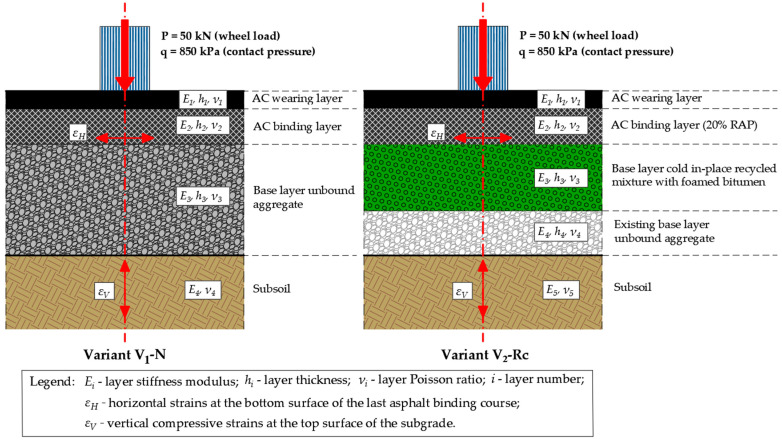
Scheme for the mechanistic design according to the elastic layer model in the elastic half-space for the proposed variants of road pavement reconstruction.

The empirical–mechanistic method was used to demonstrate the practical application of reclaimed materials to the base layer and the binding surface of the road section (variant V_2_-Rc) and to compare its fatigue life with the basic solution, including the construction of structural layers from virgin materials (variant V_1_-N). The calculation diagrams of the structure are illustrated in Figure 11.

The fatigue life of the pavement structure of the road section in question was calculated using the new fatigue criteria used in Poland (Formulas (2)–(5)). For each of the pavement layers, material constants were adopted in accordance with the catalog [33]. The calculations were made on the assumption that the pavement structure model is a multi-layered elastic half-space. The fatigue life of the asphalt layers due to cracks and due to the appearance of structural ruts was thus determined according to Criterion (6):

(6) $$\varepsilon_{V}=\;{k(1/N_{p})}^{m}$$

where $\varepsilon_{V}$ —vertical compressive strains at the top surface of the subgrade, *k* = 1.05 × 10^−2^; *m* = 0.223 (coefficient according to Chevron); *N_p_*—number of loads until critical structural deformation of the pavement (12.5 mm).

Guided by the provisions of national guidelines [66], the calculations took into account the minimum allowed asphalt *V_b_* and the maximum allowed air void content $V_{a}$ in the mixture for bituminous layers (wearing and binding).

The results of the strain calculations of the analyzed pavement structures with the characteristics and properties of the materials are presented in Table 5 for the V_1_-N variant and Table 6 for the V_2_-Rc variant. The characteristics of the asphalt layers are representative of design dynamic stiffness modulus measured at an equivalent temperature of 13 °C and 10 Hz, as assumed in national guidelines [33].

Fatigue life calculations for V_2_-Rc variant (Table 6) were additionally performed for three assumed values of the stiffness modulus E of the CIR-FB base layer to evaluate the sensitivity of the structural response to material rigidity:

- E-design = 1500 MPa—adopted as the recommended performance characteristic for RAP/crushed-stone cold recycled bases according to [74] and consistent with default assumptions in the national pavement design catalog [33];
- E-minReq = 4500 MPa—representing the minimum stiffness requirement for cold-recycled base layers applied under local Central-Eastern European climatic conditions [75];
- E-lab = 5250 MPa—corresponding to the laboratory-determined *IT-CY* stiffness modulus E for the selected final CIR-FB mixture (C/FB = 2.0/2.5%).

This approach allowed the fatigue analysis to capture the influence of actual mixture performance while preserving comparability with standardized design assumptions.

Based on the material characteristics and properties summarized in Table 5 and Table 6, the predicted fatigue life results were assumed to be (7)

(7) $$N_{100}=\min\left\{N_{f},N_{p}\right\}$$

that is,

- A total of 686,774 ESAL 100 kN for variant V_1_-N;
- A total of 2,100,781 ESAL 100 kN for the variant V_2_-Rc (E-design);
- A total of 10,993,219 ESAL 100 kN for the variant V_2_-Rc (E-minReq);
- A total of 14,254,185 ESAL 100 kN for the variant V_2_-Rc (E-lab);

where $N_{100}$ —predicted fatigue life for pavement (ESAL 100 kN); $N_{f}$ —fatigue life due to bottom-up cracking (ESAL 100 kN); and $N_{p}$ —fatigue life based on permanent deformation limit (ESAL 100 kN).

**Figure 12:**
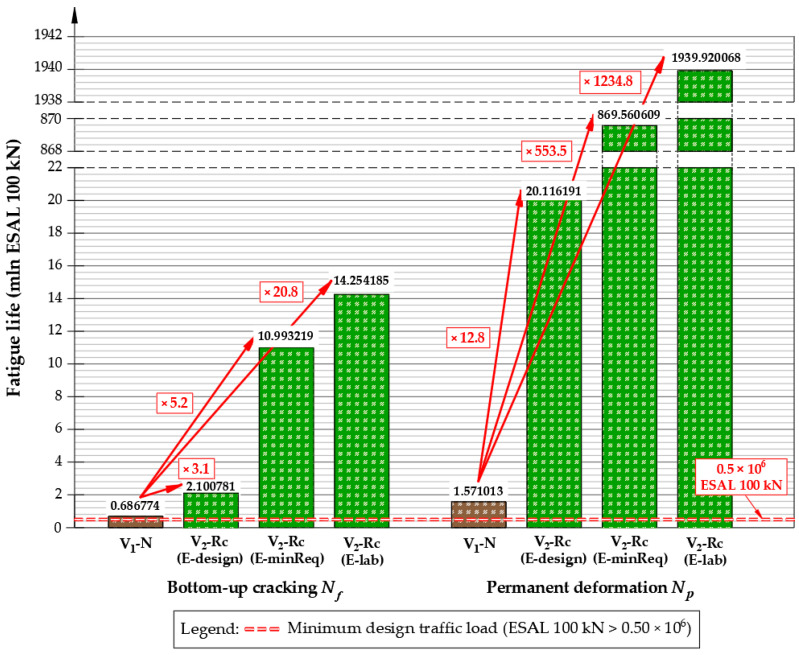
Comparison of the predicted fatigue life of road pavement for the proposed reconstruction variants.

The results of the fatigue life calculations for comparison are illustrated in Figure 12. The results demonstrated that the fatigue life of the reinforced pavement meets the requirements for the minimum designed traffic load ESAL_100kN_ > 0.50 × 10^6^. The comparative results clearly show that adopting the CIR-FB base layer (variant V_2_-Rc) substantially extends pavement fatigue life relative to the conventional V_1_-N design. The reference virgin-material variant V_1_-N reached a fatigue life of 686,774 ESAL 100 kN, serving as a baseline for comparison. When CIR-FB technology was introduced (variant V_2_-Rc), a substantial improvement in structural durability was observed.

Under the design stiffness modulus assumption (E-design = 1500 MPa), the fatigue life increased to 2,100,781 ESAL 100 kN, representing roughly a 3.1-fold extension relative to V_1_-N. When recalculated using the minimum required modulus (E-minReq = 4500 MPa), the predicted fatigue resistance rose sharply to 10,993,219 ESAL 100 kN, indicating a more than 5.2-fold improvement over the conventional reconstruction approach. The most favorable result was obtained when the laboratory-measured modulus of the optimized CIR-FB mixture (E-lab = 5250 MPa) was used as an input parameter, yielding 14,254,185 ESAL 100 kN, which corresponds to an almost 20.8-fold increase in fatigue life relative to the V_1_-N variant. For the permanent deformation criterion, the improvement is even more pronounced, with gains of about 13 (E-design), 554 (E-minReq) and 1235 times (E-lab). These gains are consistent with the marked reduction in horizontal tensile strains at the bottom of the asphalt layers and vertical compressive strains at the top of the subgrade for the V_2_-Rc structures.

The use of the V_2_-Rc variant—characterized by a higher stiffness modulus than the V_1_-N variant— resulted in a substantial reduction in structural strains. Relative to the conventional structure, horizontal tensile strains at the bottom of the asphalt layer were reduced by approximately 48–84%**,** while vertical compressive strains at the subgrade level decreased by approximately 22–49%**,** depending on the stiffness modulus adopted for the CIR-FB layer (E-design, E-minReq, E-lab). Such strain reduction directly translates into slower accumulation of fatigue damage and delayed rutting development, explaining the significant increase in fatigue life observed for the V_2_-Rc variants. As a consequence, longer maintenance intervals and lower rehabilitation frequency can be expected.

These findings clearly demonstrate that the recycling-based V_2_-Rc pavement—despite identical total thickness—provides markedly higher operational durability than the virgin-material V_1_-N structure. Performance improvement results not only from lower strain response under loading but also from enhanced stiffness and improved structural continuity of the CIR-FB layer. This advantage carries direct long-term performance, economic and environmental benefits.

### 3.4. Quantification of Material Demand and Transport Work

Both reconstruction variants were analyzed assuming an equal total structural thickness of 370 mm; however, they differ fundamentally in material demand and logistics. The recycling-based V_2_-Rc variant allowed a significant reduction in virgin-aggregate usage and minimized transport operations.

Material quantities generated during demolition and required for reconstruction for a 1 km road section (wearing, binding and base layers) are summarized in Appendix B (Table A1 and Table A2). The calculations for the V_2_-Rc variant were performed using the final CIR-FB mixture composition presented in Table 4 (Section 3.2).

**Figure 13:**
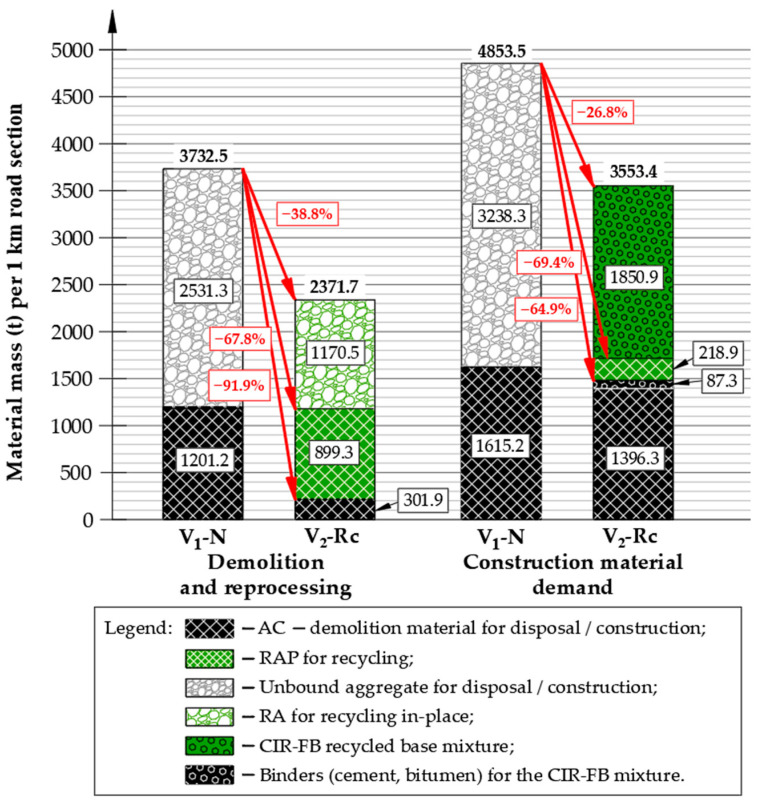
Comparison of demolition material quantities and construction material demand for the reconstruction variants per 1 km road section.

Figure 13 presents a comparative breakdown of material demand for both reconstruction approaches, including asphalt mixtures, unbound aggregate, RAP/RA contributions, and hydraulic/bituminous binders. The chart also illustrates the relative differences between variants, expressed as percentage reductions in demolition mass and new material demand achieved through in-place recycling.

The scale of demolition removal and the mass of new and reclaimed materials required directly determine transport intensity. The number of HGV transport trips (25 Mg capacity) needed to export demolition material and import construction material—including binder supply for CIR-FB production—is summarized in Table 7.

Execution of the V_1_-N variant requires full removal of existing layers (≈3700 Mg), transported off-site by approximately 151 HGV trips, followed by delivery of approximately 4850 Mg of virgin material requiring approximately 195 additional trips. This leads to high natural-aggregate demand, extended construction duration and increased environmental emissions (CO_2_, NO_x_, noise and particulate matter).

In contrast, the V2-Rc variant incorporates approximately 33% RAP and reclaimed aggregate in the CIR-FB base, plus a 20% RAP in the binding layer, eliminating ~2070 Mg of virgin material use and reducing heavy-transport frequency by approximately 74%. In total, V_2_-Rc requires 90 truckloads vs. 346 for V_1_-N—approximately 3.8 times fewer vehicle movements, resulting in substantially lower disturbance to the surrounding road network and lower pavement-wear effects.

The base course remains the most material-intensive component—accounting for 67% of the total structural thickness in V_1_-N versus 55% in V_2_-Rc (excluding the preserved 100 mm residual layer). This corresponds to >2500 Mg of aggregate demolition waste in V_1_-N, compared with partial in-place re-use in V_2_-Rc.

In the V_2_-Rc variant, total demolition and processing volume were approximately 38.8% lower compared with V_1_-N. Importantly, about 92% of the existing material was recovered and reused, instead of being removed as waste. The total mass of construction materials required for V_2_-Rc was approximately 27% lower, and about 58% of the new layer system was produced using reclaimed materials. As a result, virgin aggregate consumption decreased by roughly 65% compared with V_1_-N, providing clear environmental and economic benefits.

To evaluate the impact of construction logistics on the surrounding network, transport work was calculated for each pavement layer (wearing, binding and base), expressed in vehicle–kilometers and ton–kilometers. Detailed numerical values are summarized in Table A3 and Table A4 (Appendix B).

**Figure 14:**
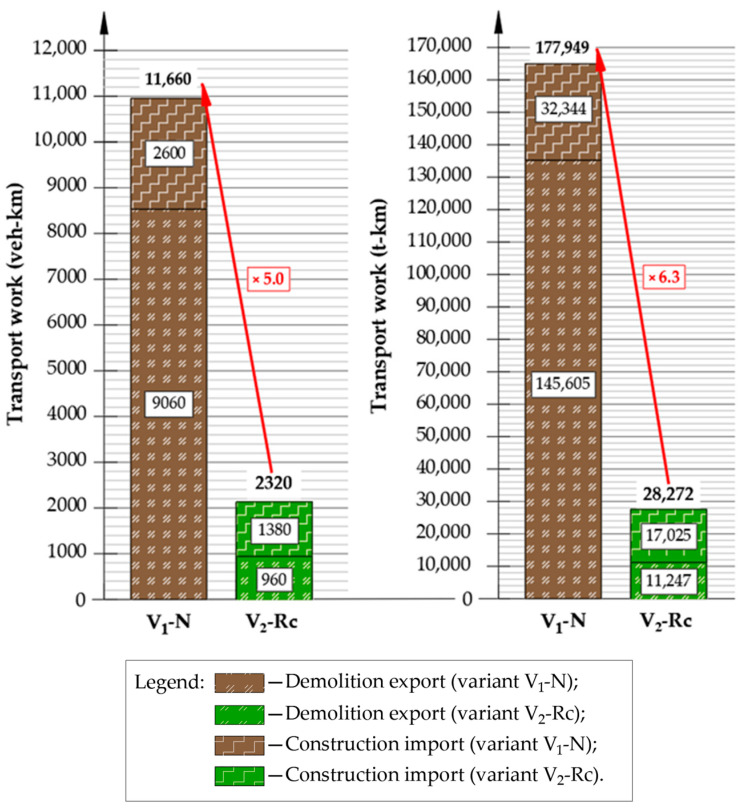
Comparison of transport work associated with export of demolition material and import of construction materials (per 1 km section).

Figure 14 compares overall transport work requirements between variants, showing the magnitude of reduction in V_2_-Rc.

Transport-work results reinforce the advantage of V_2_-Rc. For the V_1_-N variant, demolition material hauling generated approximately 9060 vehicle–km and 145,605 ton–km—which is 8100 vehicle–km and 134,358 ton–km more than under V_2_-Rc. Transport associated with the delivery of construction materials also favored V_2_-Rc, with differences of 2340 vehicle–km and 28,272 ton–km in comparison to V_1_-N.

Overall, the transport work required in V_1_-N is roughly five times higher in veh-km and 6.3 times higher in t-km than in V_2_-Rc, translating to higher fuel use, emissions, roadway distress and operational cost.

### 3.5. Construction-Phase Life Cycle Assessment

A cradle-to-gate life cycle assessment was carried out in line with ISO 14040/14044 [61,62] principles in order to quantify GHG emissions generated during pavement reconstruction works. The analysis focused on the global warming potential (GWP) expressed as kg CO_2_-equivalent (CO_2_-eq). The system boundary covered all processes occurring up to the completion of the new pavement structure, including the following:

- Production of construction materials (A1–A3);
- Transport of demolition material and inbound construction materials (A4);
- On-site works comprise the following (A5):
  ○ Milling and removal of existing asphalt layers;
  ○ Removal of existing unbound aggregate base;
  ○ Landfilling of waste asphalt concrete/aggregate;
  ○ Subgrade profiling/earthworks;
  ○ In-place recycling operations (CIR-FB);
  ○ Construction of the unbound aggregate base layer (V_1_-N) or recycled base layer (V_2_-Rc);
  ○ Placement, spreading and compaction of new asphalt layers.

GHG emissions were calculated as material mass multiplied by emission factors sourced from ecoinvent v3.12 and the ICE Database v4.1, supplemented with literature values for civil-works operations [59,60,76,77,78,79]. RAPs and RAs originating from the existing structure were modeled with zero upstream burden, as recommended for closed-loop pavement recycling systems. For the AC16 binding layer incorporating a 20% RAP, a reduced emission intensity was assumed, approximately 17% lower than that of a conventional asphalt mixture composed entirely of virgin materials. This reduction reflects both the decreased demand for new aggregate and binder, as well as the additional but limited energy required to condition RAP for reuse through cold dosing. Input material quantities and transport work values used in the calculation were taken directly from the inventory datasets presented in Table A1, Table A2 and Table A3 (Appendix B). The emission factors (EFs) adopted in the LCA calculation were compiled and are presented in Appendix C.

The calculated GHG emissions for both pavement reconstruction variants were compiled and are presented in Table A6 (Appendix C), where results are disaggregated by life-cycle stage (A1–A5), including material production, demolition processes, construction of new pavement layers and transport operations.

**Figure 15:**
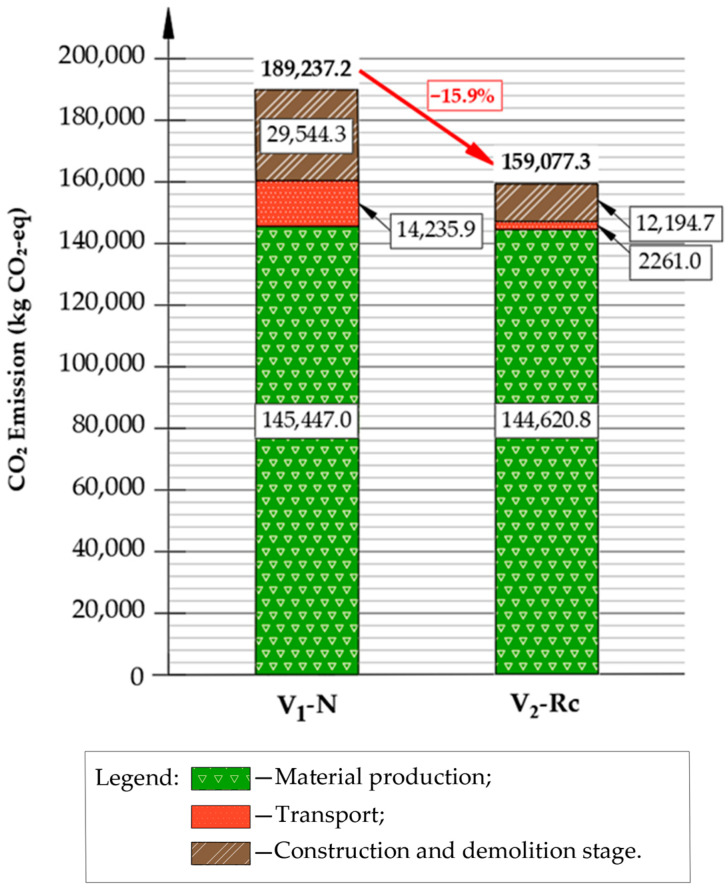
Comparison of total CO_2_-eq emissions for both pavement reconstruction variants (per 1 km section).

Figure 15 presents a comparative summary of the total kg CO_2_-eq emissions for both pavement reconstruction variants.

The life-cycle assessment results demonstrate clear differences in the carbon footprint of the two pavement reconstruction strategies. As summarized in Table A6 and illustrated in Figure 15, the total CO_2_-eq emission for the conventional variant V_1_-N reached 189,237 kg CO_2_-eq, whereas the recycling-based solution V_2_-Rc generated 159,077 kg CO_2_-eq per 1 km road section. This corresponds to an approximate 15.9% reduction in GHG emissions relative to the reference design (V_1_-N). The results, therefore, indicate a measurable environmental benefit associated with the integration of reclaimed materials and cold in-place recycling.

The dominant emission source for V_1_-N is material production (A1–A3), accounting for 145,447.0 kg CO_2_-eq, which represents approximately 77% of total emissions. This high impact results primarily from the use of virgin unbound aggregate and the full production of asphalt concrete layers. In contrast, the V_2_-Rc variant significantly reduces material-stage emissions by eliminating the need for new aggregate in the base layer and by partially substituting virgin binder with RAP. However, despite this reduction in primary aggregate demand, the overall material-stage emissions in V_2_-Rc remain comparable to V_1_-N because cement and bitumen exhibit substantially higher emission intensities per metric ton than mineral aggregate. As a result, A1–A3 emissions for V_2_-Rc reach 144,620.8 kg CO_2_-eq, only slightly lower than V_1_-N, yet achieved with substantially reduced consumption of virgin materials.

Clear differences between variants are also observed in the construction and demolition phase (A5). In V_1_-N, milling of asphalt layers and removal of the unbound base generate 29,544.3 kg CO_2_-eq, whereas in V_2_-Rc emissions fall to 12,194.7 kg CO_2_-eq, since only the wearing and binding layers require removal and a large portion of existing aggregate and asphalt is reused in situ. Waste disposal further amplifies this contrast: end-of-life landfilling contributes 9331.3 kg CO_2_-eq in V_1_-N, compared with only 754.8 kg CO_2_-eq in V_2_-Rc, reflecting the high recovery rate in the recycling-based solution.

The V_2_-Rc variant introduces one additional emission component—CIR-FB production and placement—which contributes a 5.55 t CO_2_-equivalent. Despite this process requirement, the total construction-stage emissions remain substantially lower compared with V_1_-N (12.2 vs. 29.5 t CO_2_-eq), confirming the environmental effectiveness of in-place cold recycling. Transport-related emissions also strongly favor V_2_-Rc. Due to the lower delivery of virgin materials to the site and the substantially smaller waste stream, A4 emissions decrease from 14,235.9 kg CO_2_-eq (V_1_-N) to 2261.0 kg CO_2_-eq (V_2_-Rc), representing a reduction of nearly 84%, as illustrated in Figure 15.

**Figure 16:**
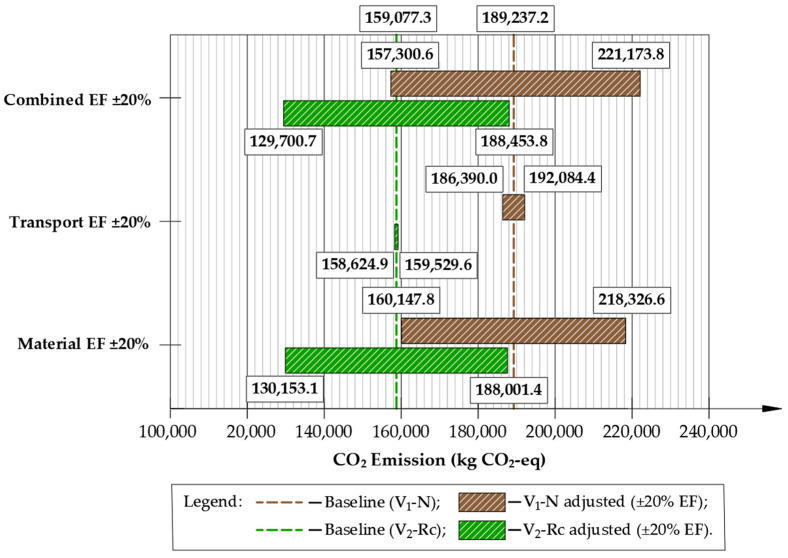
Sensitivity analysis of construction-phase CO_2_-eq results for V_1_-N and V_2_-Rc under ±20% variation in material EFs, transport EFs and combined scenarios (per 1 km section).

To assess the robustness of the LCA results, a sensitivity analysis was performed in which cradle-to-gate material emission factors (A1–A3) and transport intensity (A4) were varied by ±20%, reflecting typical uncertainty ranges reported for background LCA datasets. In addition to single-parameter scenarios (±20% materials; ±20% transport), a combined case was evaluated where both parameters were simultaneously scaled by ±20%, providing lower- and upper-bound estimates of total GHG emissions. The resulting impact on CO_2_-eq emissions for both pavement reconstruction variants is illustrated in Figure 16.

A sensitivity analysis was applied to material production (A1–A3) and transport emissions (A4) since these categories exhibit the highest documented variability in pavement LCA datasets and therefore have the greatest influence on total CO_2_-eq outcomes. Construction-stage emissions (A5) were kept constant, as equipment energy demand and on-site operation factors are significantly less variable and contribute proportionally less to the overall footprint.

The sensitivity analysis (Figure 16) demonstrated that material production (A1–A3) is the dominant contributor to total GHG emissions and the most influential variable in both pavement reconstruction variants. A 20% decrease in material emission factors reduced total emissions from 189,237 to 160,148 kg CO_2_-eq for the conventional design (V_1_-N) and from 159,077 to 130.153 kg CO_2_-eq for the recycling-based design (V_2_-Rc), corresponding to reductions of 15.4% and 18.1%, respectively. Increasing material emission factors by 20% elevated total emissions to 218,327 kg CO_2_-eq (V_1_-N) and 188,001 kg CO_2_-eq (V_2_-Rc), yet the relative difference between alternatives remained nearly unchanged.

Variations in transport intensity produced a noticeably smaller response. A ±20% change shifted total emissions only to 186,390–192,084 kg CO_2_-eq for V_1_-N and 158,306–159,211 kg CO_2_-eq for V_2_-Rc, representing differences of roughly 2–3% from the baseline. This confirms that transportation is not a primary driver of overall emissions for this case study.

When material and transport parameters were varied simultaneously, the full uncertainty envelope widened, ranging from 157,301 to 221,174 kg CO_2_-eq for V_1_-N and 129,701 to 188,454 kg CO_2_-eq for V_2_-Rc per 1 km section. Critically, V_2_-Rc remained lower in every scenario, including the worst-case (+20% materials +20% transport), where it still achieved approximately a 15% lower footprint than the conventional V_1_-N reconstruction.

### 3.6. Construction-Phase Life Cycle Cost Assessment

A construction-phase LCCA was performed to evaluate the economic performance of the two pavement reconstruction alternatives. The calculation was based on locally observed market prices obtained from regional contractors and procurement databases, used primarily to establish a consistent comparative basis between variants. All monetary values are expressed in euros per 1 km pavement section.

Cost estimation was carried out by multiplying material quantities, work volumes and transport demand (expressed in veh-km) by corresponding unit prices. Input quantities used in the calculations were taken directly from Table A1, Table A2 and Table A3 (Appendix B), ensuring full traceability between inventory data and resulting cost outputs. The complete numerical results of the LCCA are summarized in Table A7 (Appendix D).

To maintain clarity and facilitate comparison, the LCCA results were structured into three expenditure groups:

- C1—material supply costs, including virgin asphalt mixtures, bitumen, cement and unbound aggregate in V_1_-N, and AC with 20% RAP plus the CIR-FB base in V_2_-Rc;
- C2—transport and logistics, covering delivery of materials, transport of reclaimed material and disposal of demolition waste;
- C3—construction and site operations, including milling, demolition, CIR-FB mixing and placement, asphalt paving and compaction, and subgrade reshaping.

**Figure 17:**
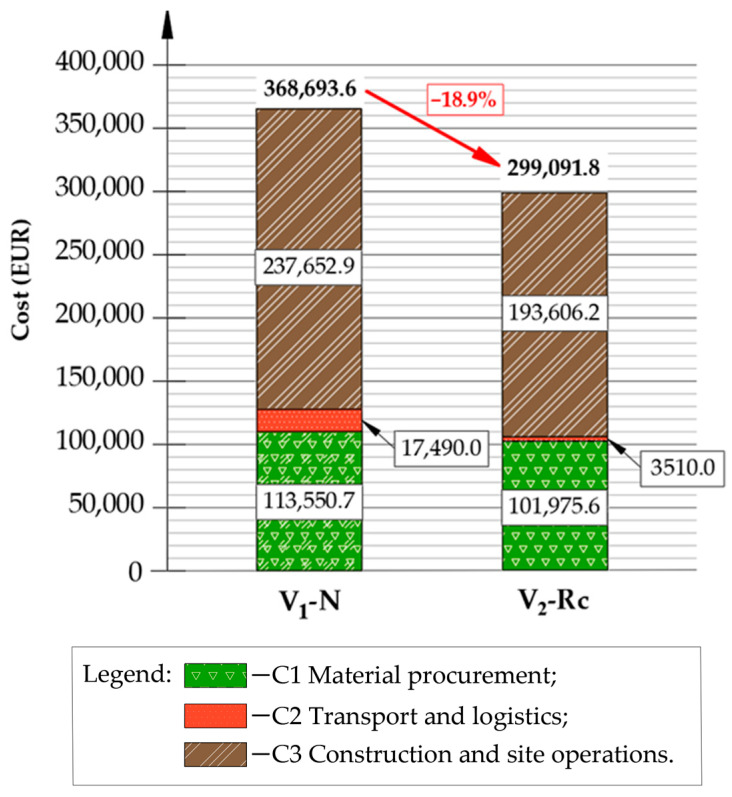
Comparison of construction-phase LCCA results for both pavement reconstruction variants (per 1 km section).

The total construction-phase cost for each variant was obtained as the sum of C1, C2 and C3. A comparative cost breakdown is illustrated in Figure 17, highlighting differences in cost structure between the conventional alternative (V_1_-N) and the recycling-based alternative (V_2_-Rc).

The construction-phase LCCA confirms the clear economic advantage of the recycling-oriented design. The total cost of variant V_2_-Rc is 299,091.8 EUR, which represents an 18.9% reduction relative to the conventional reconstruction approach V_1_-N (368,693.6 EUR per 1 km section). This result demonstrates that cold in-place recycling with foamed bitumen reduces project-scale expenditure while maintaining equivalent structural function.

The most pronounced difference between alternatives occurs in transport and logistics. Due to on-site processing of reclaimed asphalt and aggregate, V_2_-Rc requires only 3510 EUR for hauling operations, compared with 17,490 EUR in the conventional solution—a reduction exceeding 80%. This economic trend closely mirrors the LCA outcome, where transport-related emissions were also largely diminished in the recycling variant.

Material procurement (C1) remains a major cost component in both cases, though the composition differs substantially. V_1_-N relies heavily on AC layers and virgin aggregate, whereas V_2_-Rc replaces the granular base entirely with a CIR-FB layer and incorporates 20% RAP in the binding layer. Despite the cost associated with cement and bitumen required for CIR-FB production, overall material expenditure remains lower in V_2_-Rc, providing meaningful financial savings at the procurement stage.

Construction and site operations (C3) represent the largest expenditure group in both designs, dominated by asphalt placement activities. However, the elimination of full base reconstruction and significantly reduced landfill fees allows V_2_-Rc to maintain a lower total cost even when accounting for the CIR-FB process. In practice, the reduction in virgin aggregate demand and the minimized waste stream are the primary drivers of economic benefit.

Overall, the LCCA results demonstrate that the V_2_-Rc alternative delivers a robust financial benefit at the construction stage. Reduced material procurement, minimized transport demand, and limited disposal requirements collectively reinforce the economic feasibility of cold in-place recycling.

**Figure 18:**
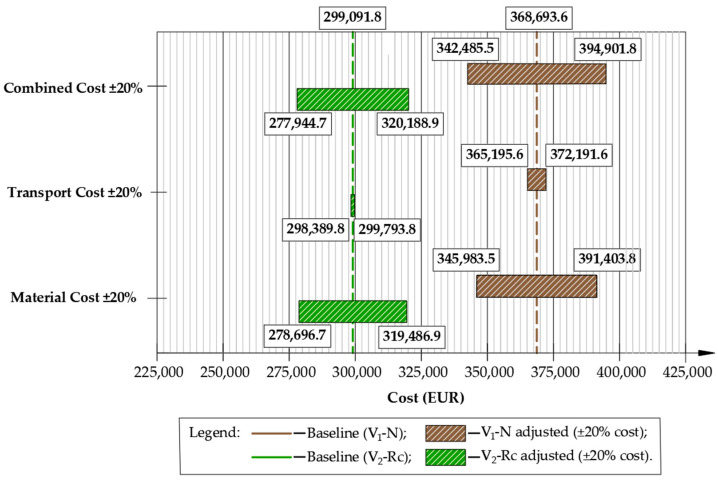
Sensitivity analysis of construction-phase LCCA results for V_1_-N and V_2_-Rc under ±20% variation in material cost, transport cost and combined scenarios (per 1 km section).

To evaluate the robustness of the LCCA outcomes and quantify the effect of economic uncertainty, a sensitivity analysis was carried out by varying unit cost rates for material procurement (C1) and transportation (C2) by ±20%. In addition to single-parameter modifications, a combined scenario was considered where both cost groups were simultaneously adjusted by ±20% to define upper- and lower-bound cost envelopes for each reconstruction strategy. Sensitivity was applied to C1 and C2 as these categories exhibit the largest market-driven price volatility (raw materials, fuel-indexed transport). C3 was not varied, since construction-stage costs are less sensitive to price fluctuations and the technological difference between variants primarily affects operation type rather than unit pricing. The results of the sensitivity evaluation are presented in Figure 18.

As presented in Figure 18, the V_2_-Rc variant remains more economical in every pricing scenario tested. When material costs decrease, both variants become cheaper, yet V_2_-Rc still delivers approximately 19% lower expenditure compared to V_1_-N. When material prices rise, the advantage persists, remaining within an 18–19% margin. This confirms that the cost gap between solutions is not sensitive to typical market swings in asphalt, binder or aggregate pricing.

Variations in transport cost have an even smaller effect on total expenditure, particularly in the recycling design. While V_1_-N responds noticeably to fuel-indexed transport changes, the V_2_-Rc alternative shifts by less than 2% because transported volumes are minimal. Under both −20% and +20% transport scenarios, the recycling variant remains approximately 18–20% cheaper than the conventional one.

The combined ±20% case produces the widest uncertainty envelope, yet the cost advantage of V_2_-Rc remains practically constant (~18–19% reduction). This stability indicates that cold in-place recycling is financially robust, even if raw-material or fuel prices increase substantially.

## 4. Discussion

### 4.1. Engineering Performance

The results indicate that the cold in-place recycling with foamed bitumen (V_2_-Rc) can successfully replace the full-depth reconstruction variant using virgin materials (V_1_-N). At equal pavement thickness, the CIR-FB base reached a stiffness modulus of approximately 5.25 GPa, which falls within the typical range reported for cold-recycled foamed-bitumen base mixtures, as documented in laboratory and field investigations [80,81,82]. The mechanistic–empirical prediction further showed that V_2_-Rc achieved 3–21 times longer fatigue life than the V_1_-N structure, depending on climatic and traffic inputs. Comparable fatigue improvements have been reported for RAP-rich cold-recycled mixtures when gradation and binder content are correctly balanced [83,84].

In addition to stiffness-based assessments, the laboratory characterization confirmed that the selected binder combination (FB = 2.5%, C = 2.0%) provided adequate cohesion and moisture resistance. The *ITS_d_*, *ITS_w_* and *TSR* results met the national and international acceptance criteria [49,75] for cold-recycled mixtures, indicating that the cement addition was sufficient to ensure early-age strength and moisture durability without excessively mixing the mixture. This balance in stabilizer content contributed directly to achieving the laboratory modulus of 5.25 GPa and, consequently, the substantial fatigue-life gains observed in the mechanistic–empirical analysis.

A more detailed analysis of stiffness inputs shows that the structural response of the V_2_-Rc variant is strongly controlled by the modulus of the CIR-FB layer. Using the catalog design value (E-design = 1500 MPa), the predicted fatigue life reached 2.10 × 10^6^ ESAL 100 kN (≈3.1 times V_1_-N). With the minimum required modulus (E-minReq = 4500 MPa), fatigue resistance increased to 1.10 × 10^7^ ESAL 100 kN (≈5.2 times V_1_-N). The highest performance was achieved with the laboratory-measured modulus (E-lab = 5250 MPa), yielding 1.43 × 10^7^ ESAL 100 kN and an almost 20.8-fold increase over the conventional design. Permanent deformation results showed even larger improvements—about 13×, 554× and 1235× for E-design, E-minReq and E-lab, respectively. These improvements reflect the marked reduction in tensile strain at the bottom of the asphalt layers and compressive strain at the subgrade, confirming the favorable stress redistribution provided by the CIR-FB layer.

Previous investigations demonstrated that semi-bound foamed-bitumen layers reduce tensile strain concentration under asphalt layers, mitigating fatigue cracking and rutting [74]. Similarly to the present study, recent mechanistic–empirical and field investigations have shown that well-designed CIR base layers can achieve structural and fatigue performance comparable to conventional asphalt base alternatives on low- and medium-volume roads [85,86]. These correlations suggest that the high fatigue reserve observed for V_2_-Rc provides sufficient structural tolerance to RAP variability and freeze–thaw conditions typical for the region.

A key contribution of the present study is the demonstrated link between mixture composition (cement/foamed-bitumen content), stiffness modulus, fatigue life and CO_2_ impact. Higher stabilizer content increases stiffness, but also increases embodied emissions—confirming non-linear optimization behavior also observed by [14,78].

The selected composition (FB = 2.5%, C = 2.0%) provided fatigue capacity greatly exceeding design traffic without over-specifying binder content. This follows the design-decision logic proposed in pavement LCA frameworks [78,87], confirming its applicability for municipal-scale rehabilitation.

### 4.2. Environmental and Economic Performance

The LCA results clearly showed lower carbon emissions for V_2_-Rc, attributed mainly to lower production of virgin aggregate, reduced asphalt binder demand and a significant decrease in transport work. Similar reductions, often in the range of 10–50%, due to RAP use and cold-recycling technologies have been reported by [77,88].

The material-flow assessment confirms the efficiency of V_2_-Rc, which reduced demolition waste by approximately 39% and enabled reclaimed materials to cover approximately 58% of construction demand, substantially lowering the need for virgin aggregate. This reduction in virgin-resource consumption not only decreases the carbon intensity of the reconstruction process but also contributes to the preservation of natural aggregate deposits and reduces land-use pressure associated with quarrying. Thus, the environmental benefits of V_2_-Rc arise both from avoided emissions and from avoided raw-material depletion, reinforcing the overall sustainability advantage of the recycling-based solution.

For a 1 km road section, total GHG emissions decreased from 189,237 to 159,077 kg CO_2_-eq, corresponding to a 15.9% reduction relative to V_1_-N. These savings arise from minimized binder production, reduced virgin aggregate demand and significantly lower transport intensity. LCCA further confirmed that construction cost decreased from 368,693.6 to 299,091.8 EUR (≈18.9%), and the cost advantage of V_2_-Rc remained stable (~18–19%) across ±20% material-price sensitivity scenarios.

The LCCA results confirmed that V_2_-Rc is more economical during the construction stage despite requiring specialized CIR equipment. Savings in aggregate supply, transport and waste disposal led to an overall reduction in direct cost, aligning with trends reported in economic assessments of RAP- and CIR-based road rehabilitation [77,89]. Sensitivity analysis confirmed that the cost–benefit advantage persists even under ±20% price variation.

This jointly supports the conclusion that higher structural durability does not conflict with economic or environmental performance—the recycling-based variant maximizes structural life while minimizing emissions and material extraction.

### 4.3. Practical Implications, SWOT Perspective and Research Needs

The complementary SWOT analysis (Appendix E) synthetically summarizes practical implementation aspects that are not captured by numerical indicators. The conventional variant V_1_-N is based on well-known technologies and entails relatively low implementation risk, but it requires large quantities of virgin materials, generates substantial waste and involves intensive construction traffic. In contrast, the recycling-based variant V_2_-Rc aligns with circular economy principles by maximizing in situ reuse of existing layers and reducing extraction, transport and landfill demand, at the cost of higher requirements for equipment, planning and quality control of reclaimed materials.

The SWOT summary highlights that V_2_-Rc reduces excavation, landfilling and transport impacts, while requiring stricter control over RAP composition—observations consistent with high-RAP mix reviews and implementation studies [90,91]. 

Future research should extend the present analysis beyond the construction stage to include the use, maintenance and end-of-life phases, and should validate the predicted fatigue performance through long-term field monitoring. Additional work is also needed to quantify the effects of RAP variability, aging and moisture conditioning on CIR-FB behavior and to further refine transfer functions used in mechanistic–empirical design for cold recycled base layers.

## 5. Conclusions

This study compared two pavement rehabilitation strategies for a municipal road: V_1_-N—full reconstruction using virgin materials, and V_2_-Rc—cold in-place recycling with foamed bitumen (CIR-FB) and reclaimed materials (RAP and RA). The evaluation integrated a mechanistic–empirical design, laboratory characterization of CIR-FB mixtures (stiffness modulus ≈ 5.25 GPa, binder content FB = 2.5% and C = 2.0%), material balance, environmental LCA, economic LCCA and SWOT interpretation.

The following conclusions summarize the pavement performance, environmental, economic, and implementation outcomes of the study.

- Laboratory testing confirmed a stiffness modulus of approximately 5.25 GPa for the CIR-FB base. Incorporating this measured value into mechanistic–empirical calculations enabled fatigue life prediction using realistic material behavior rather than catalog assumptions. The resulting structure achieved fatigue life between approximately three and over twenty times higher than V_1_-N at equal thickness, confirming the performance potential of CIR-FB for municipal pavements with light-to-medium traffic loading.
- Material balance analysis showed that V_2_-Rc reduced the need for virgin materials by approximately 27% and lowered demolition waste by around 39%. In total, 92% of the existing pavement was reused in situ, decreasing aggregate extraction and supporting preservation of natural mineral resources.
- Transport demand was significantly lower in V_2_-Rc: the number of truck operations decreased nearly fourfold, total vehicle–kilometers by about fivefold and ton–kilometer by more than sixfold. This reflects clear reductions in energy consumption, construction logistics and transport-related emissions.
- LCA results confirmed that the recycling-based variant generated a substantially lower carbon footprint due to reduced binder production, lower virgin aggregate demand and shorter haul distances. LCCA showed that V_2_-Rc offered lower construction-stage agency cost, with its economic advantage remaining robust under ±20% price variation. Quantitatively, for a 1 km road section, total CO_2_ emissions decreased from 189,237 to 159,077 kg CO_2_-eq (≈15.9%), while reconstruction cost was reduced by approximately 19%, with the economic advantage remaining stable (~18–19%) under ±20% price-sensitivity scenarios.
- The combined results demonstrate that extended pavement performance can be achieved without increased environmental or economic burden. The V_2_-Rc variant delivered longer fatigue life while simultaneously reducing emissions, resource use and transport intensity.
- A link was identified between mixture composition (cement and foamed-bitumen content), stiffness modulus, fatigue resistance and emission intensity. Although increasing stabilizer content improved structural response, it also elevated embodied energy, confirming that mixture optimization requires balance rather than maximization of binder dosage.
- The V_2_-Rc variant is aligned with circular economy practice and is feasible for municipal networks, provided that reclaimed material quality is consistently controlled during construction.
- The developed approach—integrating performance modeling, LCA, LCCA and SWOT—may serve as a transferable framework for local authorities seeking to reduce virgin resource dependency while maintaining required pavement performance.

These findings confirm the initial research hypothesis that reclaimed materials, when properly characterized and structurally integrated, can extend pavement fatigue life while simultaneously reducing material extraction, emissions, and cost.

## Figures and Tables

**Table 1:** Summary of pavement damage types identified on the surveyed road section.

Damage Type	Count	Share (%)	Main Cause of Damage
Traverse cracks	145	40.6	Thermal stresses
Longitudinal cracks	85	23.8	Insufficient capacity/settlement
Patches	79	22.1	Previous local repairs
Alligator cracking	7	2.0	Fatigue of asphalt layers
Stripping and raveling	37	10.4	Moisture, freeze–thaw cycles, abrasion
Potholes	4	1.1	Water ingress, mechanical loading

**Table 2:** Test results of bitumen extracted from the RAP (Valid N—number of specimens, the same number for both RAP fractions).

Property	Test Method	Valid N	Mean Value	
			RAP-1	RAP-2
Penetration at 25 °C (0.1 mm)	EN 1426 [45]	10	14	16
Softening point (*T_R&B_*) (°C)	EN 1427 [46]	4	64.1	68.8

**Table 4:** Composition of the CIR-FB mixture.

Component	Quantity (kg/m^3^)	Percentage (%)
Reclaimed asphalt pavement (RAP-2)	755	35.1
Reclaimed aggregate (RA)	1299	60.4
Foamed bitumen 50/70	54	2.5
Cement CEM II/B-V 42.5 R	43	2.0
Total	2151	100.0

**Table 5:** Characteristics and material properties for the V_1_-N pavement variant with strain calculation results.

Material Characteristics			Material Properties		
Layer Number *i*	Layer Type	Layer Material	*ν_i_*	*h_i_*	*E_i_*
				(mm)	(MPa)
1	Wearing	AC 11	0.30	40	9300
2	Binding	AC 16	0.30	80	10,300
3	Base	Unbound aggregate, C_NR_	0.30	250	250
4	Subsoil	Medium sands	0.35	-	80
Strain calculations					
Horizontal deformations on the bottom surface of the last asphalt binding course (depth = 120 mm) ε_xx_ = ε_yy/_ $\varepsilon_{H}$ (με)				173.10	
Vertical compressive strains at the top surface of the subgrade (depth = 370 mm) $\varepsilon_{V}$ (με)				−524.30	

**Table 6:** Characteristics and material properties for the V_2_-Rc pavement variant with strain calculation results.

Material Characteristics			Material Properties				
Layer Number *i*	Layer Type	Layer Material	*ν_i_*	*h_i_*	*E_i_*		
				(mm)	(MPa)		
					E-Design	E-minReq	E-Lab
1	Wearing	AC 11	0.30	40	9300	9300	9300
2	Binding	AC 16 (20% RAP)	0.30	80	10,300	10,300	10,300
3	Base	CIR-FB	0.30	150	1500	4500	5250
4	Base	Existing unbound aggregate, C_NR_	0.30	100	250	250	250
5	Subsoil	Medium sands	0.35	-	80	80	80
Strain calculations							
Horizontal strains at the bottom surface of the last asphalt binding course (depth = 120 mm) ε_xx_ = ε_yy/_ $\varepsilon_{H}$ (με)					90.76	34.97	28.54
Vertical compressive strains at the top surface of the subgrade (depth = 370 mm) $\varepsilon_{V}$ (με)					−408.60	−282.50	−266.60

**Table 7:** Truck transport demand per 1 km reconstruction section.

Structure Layer	Export of Demolition Material (Trips)		Import of Construction Material (Trips)	
	Variant V_1_-N	Variant V_2_-Rc	Variant V_1_-N	Variant V_2_-Rc
Wearing	21	21	21	21
Binding	28	0	44	44
Base	102	0	130	4
Entire pavement structure	151	21	195	69

**Figure A1:**
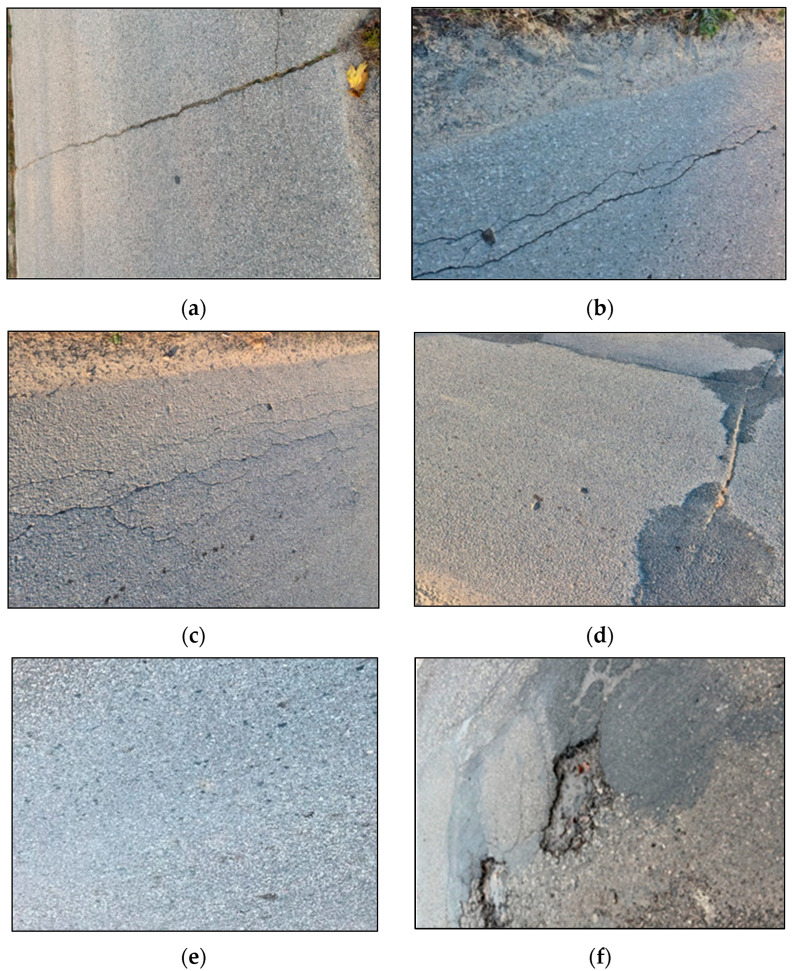
The most common damages in the assessed section: (**a**) single transverse fracture; (**b**) single longitudinal cracks; (**c**) alligator cracking; (**d**) patches; (**e**) stripping and raveling in the wearing layer; and (**f**) potholes.

**Figure A2:**
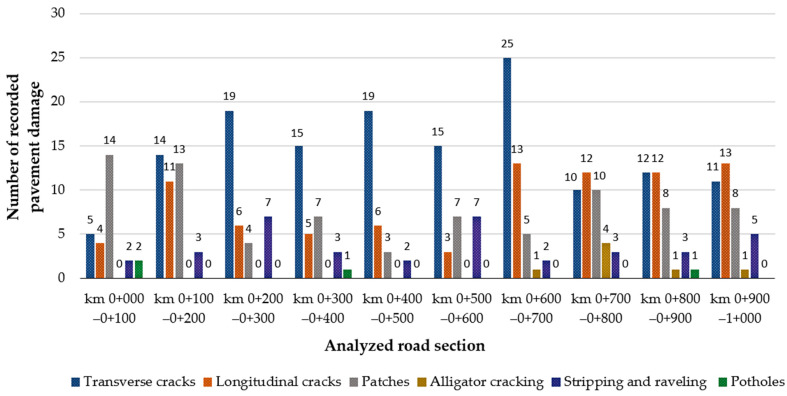
Number of pavement damages on individual road sections.

## Data Availability

The original contributions presented in this study are included in the article. Further inquiries can be directed to the corresponding author.

## Abbreviations

The following abbreviations are used in this manuscript:

RAP	Reclaimed asphalt pavement
RA	Reclaimed aggregate
CIR	Cold in-place recycling
FB	Foamed bitumen
CIR-FB	Cold in-place recycling with foamed bitumen
SWOT	Strengths, weaknesses, opportunities, and threats analysis
LCA	Life cycle assessment
LCCA	Life cycle cost assessment
MEPDG	Mechanistic–empirical pavement design guide
AC	Asphalt concrete
CBR	California bearing ratio
ESAL	Equivalent standard axle load
HGV	Heavy goods vehicle
RH	Relative humidity
OMC	Optimum moisture content
ITS	Indirect tensile strength
TSR	Tensile strength ratio
UCS	Unconfined compressive strength
IT-CY	Indirect tensile stiffness modulus
HMA	Hot mix asphalt
GHG	Greenhouse gas
GWP	Global warming potential
EF	Emission factor

## Appendix A. Visual Assessment of the Technical Condition of the Road Pavement

Figure A1 shows the most common types of damage in the analyzed road section.

Numerous pavement surface defects were identified, each associated with characteristic underlying mechanisms. Transverse cracks (Figure A1a) are primarily related to thermally induced stresses generated within the asphalt layers. Longitudinal cracks (Figure A1b) indicate insufficient bearing capacity of the pavement structure and may also reflect local, non-uniform settlement of the underlying layers. Alligator cracking (Figure A1c) is associated with inadequate structural capacity of the base or subbase and advanced fatigue deterioration of the asphalt layers. Patches (Figure A1d) represent areas that underwent previous ad hoc repairs. Stripping and raveling (Figure A1e) result from moisture infiltration, freeze–thaw cycles and traffic-induced abrasive action. Potholes (Figure A1f) originate from combined mechanical loading, water penetration and temperature variations that weaken the pavement structure.

Figure A2 shows the number of recorded damages in the consecutive hectometers of the pavement section.

A total of 357 surface distresses were recorded along the evaluated section. The analyzed hectometer segments differed substantially in the distribution, type and extent of the observed damage (Figure A2). The number of distresses ranged from 27 pieces per 100 m (section km 0 + 000–0 + 100) to 46 per 100 m (section km 0 + 600–0 + 700). This means that these are not local accidental damages, but are the result of inadequate structural capacity of the existing pavement under prevailing traffic conditions, indicating the need to rebuild the entire road section.

Transverse cracks constitute the largest share of pavement damage (41%), which is consistent with thermally induced distress mechanisms previously identified. Longitudinal cracks account for 24% of all defects and correspond to areas with reduced structural capacity or local subsidence. Patches represent 22% of the recorded distresses, reflecting locations of past repair interventions; their extensive occurrence suggests limited long-term durability of previous maintenance actions. Stripping and raveling, comprising 10% of the damage, indicate progressive surface deterioration caused by moisture and freeze–thaw cycles under traffic loading. Alligator cracking (2%) reveals localized fatigue-related weakening of the structure. Potholes account for only 1% of defects but significantly reduce pavement serviceability where they occur. Permanent deformations in the form of ruts were also observed, indicating further weakening of the pavement layers.

The lack of adequate transverse slopes, resulting from numerous cracks and raveling in the pavement, leads to inadequate water drainage. This results in stagnation of the water on the edge of the road and accelerated degradation of its structure. Numerous cracks allow water to easily penetrate the lower layers of the road structure. Freeze–thaw cycles facilitate moisture penetration into the pavement layers, which reduces bearing capacity and accelerates structural degradation, as documented in previous studies [92]. As a result, the load capacity of the entire surface deteriorates, making it necessary to react quickly with a rehabilitation intervention.

The scope of repair of the damaged pavement was determined on the basis of the provisions of the catalog [35] using the percentage of the road pavement showing surface damage (patches, cavities and cracks).

The following damage area was recorded in the examined section:

- A total of 596 m^2^—on the left lane;
- A total of 559 m^2^—in the right lane;

This is about 20.1% of the total paved area (5500 m^2^).

## Appendix B. Detailed Calculation Results for Material Quantities and Transport Work

Table A1 summarizes the quantities of materials generated from demolition for both reconstruction variants, while Table A2 presents the demand for virgin and reclaimed materials required to rebuild a 1 km road section.

The following bulk densities were used to calculate the mass of materials, based on information obtained from regional contractors and own laboratory data:

- AC wearing layer—2.35 Mg/m^3^;
- AC binding layer—2.40 Mg/m^3^;
- Unbound aggregate of the base layer—2.12 Mg/m^3^;
- Base layer CIR-FB mix—2.15 Mg/m^3^;
- Cement—3.05 Mg/m^3^;
- Bitumen—1.02 Mg/m^3^.

**Table A1:** Summary of the mass of materials obtained from the demolition of the existing road structure over a 1 km long road section.

Structure Layer	Mass of Demolition Material (Mg)		Recycled Material Mass (Mg)	
	Variant V_1_-N	Variant V_2_-Rc	Variant V_1_-N	Variant V_2_-Rc
Wearing	520.8	301.9	0.0	218.9
Binding	680.4	0.0	0.0	680.4
Base	2531.3	0.0	0.0	1170.5
Entire pavement structure	3732.5	301.9	0.0	2069.8

**Table A2:** Summary of the mass of materials required to construct pavement structure variants over a 1 km section.

Structure Layer	Mass of Virgin Materials (Mg)		Mass of Reclaimed Materials (Mg)	
	Variant V_1_-N	Variant V_2_-Rc	Variant V_1_-N	Variant V_2_-Rc
Wearing	520.8	520.8	0.0	0.0
Binding	1094.4	875.5	0.0	218.9
Base	3238.3	87.3	0.0	1850.9
Entire pavement structure	4853.5	1483.6	0.0	2069.8

Transport work was evaluated using two metrics: vehicle–kilometers and ton–kilometers, as summarized in Table A2 and Table A3. The calculations take into account the number of trips of heavy goods vehicles necessary for the transport of materials (Table 7), the weight of materials (Table A1 and Table A2) and the established distances to nearby enterprises:

- Mineral quarry—5 km;
- Asphalt plant—10 km;
- Cement plant—10 km;
- Industrial waste landfill—30 km.

**Table A3:** Transport work (veh-km) associated with the export of demolition material and import of construction material for a 1 km pavement section.

Structure Layer	Export of Demolition Material (veh-km)		Import of Construction Material (veh-km)	
	Variant V_1_-N	Variant V_2_-Rc	Variant V_1_-N	Variant V_2_-Rc
Wearing	1260	960	420	420
Binding	1680	0	880	880
Base	6120	0	1300	80
Entire pavement structure	9060	960	2600	1380

**Table A4:** Transport work (t-km), associated with the export of demolition material and import of construction material for a 1 km road section.

Structure Layer	Export of Demolition Material (t-km)		Import of Construction Material (t-km)	
	Variant V_1_-N	Variant V_2_-Rc	Variant V_1_-N	Variant V_2_-Rc
Wearing	15,624	11,247	5208	5208
Binding	32,832	0	10,944	10,944
Base	97,149	0	16,192	873
Entire pavement structure	145,605	11,247	32,344	17,025

## Appendix C. LCA Inventory and Calculation Results

Table A5 presents the emission factors used in the LCA together with the corresponding value ranges, selected calculation values and reference sources.

**Table A5:** Emission factors applied in the LCA (sources: [59,60,76,77,78,79]).

Material/Process	Emission Factor (kg CO_2_-eq/t)	
	Range	Value Used
Asphalt concrete production	50–75	70
Asphalt concrete with 20% RAP	48–60	57
Bitumen	350–450	400
Unbound aggregate	5–12	10
Cement	600–750	680
RAP used in CIR-FB base layer	0	0
RA used in CIR-FB base layer	0	0
CIR-FB in situ processing	2–4	3
Asphalt paving and compaction	2–4	3
Unbound base compaction	1–2	1.5
Milling/demolition	1.5–3	2
Subgrade profiling/earthworks	0.3–0.6	0.5
Landfilling of waste asphalt concrete/aggregate	2–3	2.5
Transport (HGV)	0.07–0.10	0.08

Table A6 presents the total CO_2_-equivalent emissions calculated for both pavement reconstruction variants (V_1_-N and V_2_-Rc), broken down by life-cycle stage.

**Table A6:** Total CO_2_-eq emissions for both design variants, broken down by lifecycle stage.

Material/Process/ Lifecycle Stage (LCA Boundary—A1–A5)	CO_2_-eq Emission (kg CO_2_-eq)	
	Variant V_1_-N	Variant V_2_-Rc
A1–A3 Material production		
HMA AC	113,064.0	36,456.0
HMA AC with 20% RAP	0.0	62,380.8
Unbound aggregate	32,383.0	0.0
RAP used in CIR-FB base layer	0.0	0.0
RA used in CIR-FB base layer	0.0	0.0
Cement used in CIR-FB base layer	0.0	27,160.0
Bitumen used in CIR-FB base layer	0.0	19,400.0
A4 Transport		
Delivery of virgin materials	2587.5	1362.0
Removal of waste materials	11,648.4	899.8
A5 Construction and demolition work		
Milling and demolition of asphalt layers	2402.4	1041.6
Removal of unbound aggregate layer	5062.6	0.0
Waste treatment/disposal	9331.3	754.8
Construction of new pavement layers:		
Wearing layer (HMA)	1562.4	1562.4
Binding layer (HMA)	3283.2	3283.2
Unbound aggregate base	4857.5	0.0
CIR-FB recycled base layer	0.0	5552.7
Subgrade profiling/earthworks	3055.0	0.0
Total emissions (A1 − A3 + A4 + A5)	189,237.2	159,077.3

## Appendix D. LCCA Calculation Results

Table A7 presents the complete LCCA calculation for both pavement reconstruction variants, including material, transport and construction activity costs for the 1 km reference section. Unit prices applied in the calculation were derived from regional contractor price lists and typical procurement rates observed in municipal road contracts. For clarity, the following assumptions should be noted when interpreting Table A7:

- AC 11 and AC 16 prices were based on standard market values for hot-mix asphalt, including supply and placement;
- The AC binding layer with 20% RAP was assigned a reduced unit cost relative to the virgin AC mixture, reflecting lower binder demand and partial replacement of new asphalt with reclaimed material;
- Unbound aggregate was priced per square meter, assuming material supply and mechanical compaction;
- Prices for cement and bitumen represent material supply costs only;
- CIR-FB construction costs were calculated as a unit rate per square meter of recycled base, based on contractor quotations for cold in-place recycling with foamed bitumen;
- Transport rates were applied per vehicle–kilometer and include fuel, loading and haulage;
- Landfill disposal fees reflect gate charges for asphalt and aggregate waste, excluding transport, which is accounted for separately.

**Table A7:** Total construction-phase LCCA costs for both design variants, broken down by cost category.

Material/Process/ Cost Stage	Cost (EUR)	
	Variant V_1_-N	Variant V_2_-Rc
C1 Material procurement		
HMA AC 11 wearing layer	28,071.1	28,071.1
HMA AC 16 binding layer	51,874.6	0.0
HMA AC 16 binding layer with 20% RAP	0.0	44,870.4
Unbound aggregate, C_NR_ 0/31.5 mm	33,605.0	0.0
Cement used in CIR-FB base layer	0.0	6025.6
Bitumen used in CIR-FB base layer	0.0	23,008.4
C2 Transport and logistics		
Delivery of virgin materials	3900.0	2070.0
Removal and hauling of waste materials	13,590.0	1440.0
C3 Construction and site operations		
Milling and demolition of asphalt layers	14,573.0	7202.0
Removal of unbound aggregate base	6567.0	0.0
Waste disposal (landfill fee)	42,549.9	5223.2
Construction activities:		
AC 11 wearing layer, 40 mm	32,550.0	32,550.0
AC 16 binding layer, 80 mm	120,384.0	0.0
AC 16 binding layer, with 20% RAP	0.0	120,384.0
Unbound aggregate base, 250 mm	16,716.0	0.0
CIR-FB recycled base layer, 150 mm	0.0	28,247.0
Subgrade profiling/earthwork	4313.0	0.0
Total life-cycle construction cost (C1 + C2 + C3)	368,693.6	299,091.8

The adopted cost values fall within representative regional price levels, including approximately 50–65 EUR/t for asphalt mixtures (material), 60–110 EUR/t including paving, 150–160 EUR/t for cement, and ~4.5–5.0 EUR/m^2^ for the CIR-FB recycling process (equipment only, excluding cement and bitumen supply). Transport was valued at 1.4–1.7 EUR per veh-km, while landfill disposal fees ranged from approximately 8–18 EUR/t depending on material type. These ranges confirm that the LCCA reflects realistic market conditions and provides a robust basis for comparing the two pavement reconstruction strategies.

## Appendix E. SWOT Analysis Results

The results of the SWOT analysis for each of the proposed variants are presented in Table A8 and Table A9.

**Table A8:** SWOT analysis for the reconstruction of the V_1_-N variant of the pavement structure (conventional approach).

Strengths	Weaknesses
Proven and well-established construction technology with predictable performance.	High consumption of virgin materials and natural aggregate.
Availability of equipment, contractors and standard procedures.	High material production and transport costs.
Low technological risk during construction.	Increased environmental impacts (emissions and resource depletion).
Long-term experience and high reliability of conventional materials.	Greater volume of waste generated during reconstruction.
Opportunities	Threats
Stable supply chains and strong market availability for conventional materials.	Rising material and fuel prices increasing total construction costs.
Potential to employ local workforce and small contractors.	Possible future restrictions on the use of nonrecycled materials.
Easy integration with existing road management systems.	Growing environmental regulations limiting traditional approaches.
Summary: A mature and predictable technology with low construction risk, but characterized by low material efficiency, high consumption of virgin resources, and a significant environmental burden.	

**Table A9:** SWOT analysis for the reconstruction of the V_2_-Rc variant of the pavement structure (sustainable approach).

Strengths	Weaknesses
High material efficiency through reuse of RAP and RA.	Requires specialized equipment and trained personnel.
Significant reduction in GHG emissions and construction waste.	Dependence on the quality and homogeneity of the reclaimed materials.
Lower construction cost and reduced implementation time due to in-place processing.	Limited recycling infrastructure in certain regions.
Improved fatigue life and deformation resistance of recycled layers.	Need for additional laboratory testing and process control.
Opportunities	Threats
Full alignment with EU circular economy and climate-neutrality policies.	Variability in the regulatory acceptance of recycled materials.
Access to national and EU funding for sustainable infrastructure projects.	Uncertainty in long-term field performance.
Promotion of innovation and regional development in road engineering.	Market hesitation due to perceived technological risk.
Summary: A modern reconstruction solution aligned with circular economy principles, offering clear benefits in durability, waste reduction and cost efficiency, while presenting moderate technological risk related to reclaimed-material variability and equipment requirements.	

## References

[1] Świtała M., Regulska K. (2023). Life Cycle Cost Analysis as a Tool for Road Infrastructure Management—Selected Issues. Roads Bridg..

[2] Gryszpanowicz P., Gasik-Kowalska N., Kacprzak M., Rymsza B. (2023). Road Investments Conducted on the Principles of a Circular Economy and Their Impact on Funding for Road Repairs and Ongoing Maintenance. Roads Bridg.—Drogi i Mosty.

[3] Mantalovas K., Di Mino G., Jimenez Del Barco Carrion A., Keijzer E., Kalman B., Parry T., Lo Presti D. (2020). European National Road Authorities and Circular Economy: An Insight into Their Approaches. Sustainability.

[4] Kamali Saraji M., Torabi M. (2025). Progress Toward a Circular Economy: A Comparative Analysis of EU Member States. Sustainability.

[5] De Abreu V.H.S., Da Costa M.G., Da Costa V.X., De Assis T.F., Santos A.S., D’Agosto M.D.A. (2022). The Role of the Circular Economy in Road Transport to Mitigate Climate Change and Reduce Resource Depletion. Sustainability.

[6] Xia X., Zhao Y., Tang D. (2025). The state-of-the-art review on the utilization of reclaimed asphalt pavement via hot in-place recycling technology. J. Clean. Prod..

[7] Tsakoumaki M., Plati C. (2024). A Critical Overview of Using Reclaimed Asphalt Pavement (RAP) in Road Pavement Construction. Infrastructures.

[8] Coelho L.M., Guimarães A.C.R., de Azevedo A.R.G., Monteiro S.N. (2024). Sustainable Reclaimed Asphalt Emulsified Granular Mixture for Pavement Base Stabilization: Prediction of Mechanical Behavior Based on Repeated Load Triaxial Tests. Sustainability.

[9] Wang J., Sha C., Ly S., Wang H., Sun Y., Guo M. (2023). Life Cycle Carbon Emissions and an Uncertainty Analysis of Recycled Asphalt Mixtures. Sustainability.

[10] (2017). Project Selection Guidelines for Cold In-Place and Cold Central Plant Recycling (Tech Brief).

[11] (2019). Foamed Bitumen Stabilised Pavements: Deformation Performance.

[12] Loizos A., Papavasiliou V. (2006). Evaluation of Foamed Asphalt Cold In-Place Pavement Recycling Using Nondestructive Techniques. J. Transp. Eng..

[13] (2022). Resource Responsible Use of Reclaimed Asphalt Pavement in Asphalt Mixtures (Tech Brief).

[14] Amarh E.A., Santos J., Flintsch G.W., Diefenderfer B.K. (2022). Evaluating the Potential Environmental Benefits of Cold Recycling-Based Methods for Flexible Pavement Rehabilitation in Virginia. Transp. Res. Rec. J. Transp. Res. Board.

[15] Gkyrtis K., Plati C., Loizos A. (2023). Structural Performance of Foamed Asphalt Base in a Full Depth Reclaimed and Sustainable Pavement. Sustainability.

[16] Zhao H., Ren J., Chen Z., Luan H., Yi J. (2021). Freeze and thaw field investigation of foamed asphalt cold recycling mixture in cold region. Case Stud. Constr. Mater..

[17] Stępień J., Maciejewski K. (2022). Using Reclaimed Cement Concrete in Pavement Base Mixes with Foamed Bitumen Produced in Cold Recycling Technology. Materials.

[18] Mrugała M. (2012). Soil Stabilization with Foamed Bitumen. Struct. Environ..

[19] Chomicz-Kowalska A., Stepien J. (2016). Cost and Eco-Effective Cold In-Place Recycled Mixtures with Foamed Bitumen during the Reconstruction of a Road Section under Variable Load Bearing Capacity of the Subgrade. Procedia Eng..

[20] Flores-Ruiz D., Montoya-Alcaraz M., García L., Gutiérrez M., Calderón-Ramírez J. (2025). Mitigation Strategies Based on Life Cycle Assessment for Carbon Dioxide Reduction in Asphalt Pavements: Systematic Review. Sustainability.

[21] Martinez-Soto A., Calabi-Floody A., Valdes-Vidal G., Hucke A., Martinez-Toledo C. (2023). Life Cycle Assessment of Natural Zeolite-Based Warm Mix Asphalt and Reclaimed Asphalt Pavement. Sustainability.

[22] Plati C., Tsakoumaki M. (2023). Life Cycle Assessment (LCA) of Alternative Pavement Rehabilitation Solutions: A Case Study. Sustainability.

[23] Siverio Lima M.S., Makoundou C., Sangiorgi C., Gschösser F. (2022). Life Cycle Assessment of Innovative Asphalt Mixtures Made with Crumb Rubber for Impact-Absorbing Pavements. Sustainability.

[24] Elnaml I., Mohammad L.N., Baumgardner G., Cooper S., Cooper S. (2024). Sustainability of Asphalt Mixtures Containing 50% RAP and Recycling Agents. Recycling.

[25] Buttitta G., Giancontieri G., Parry T., Lo Presti D. (2023). Modelling the Environmental and Economic Life Cycle Performance of Maximizing Asphalt Recycling on Road Pavement Surfaces in Europe. Sustainability.

[26] Ibrahim H., Marini S., Farina A., Lanotte M. (2024). Integrating Mechanistic-Empirical Pavement Analysis in the Life Cycle Assessment Use Phase and Monetization of Environmental Impacts to Promote Low Carbon Transportation Materials. Transp. Res. Rec. J. Transp. Res. Board.

[27] (2023). Stan dróg powiatowych i gminnych w Polsce—Raport Najwyższej Izby Kontroli 2023.

[28] Dokyi G.O., Osei K.K., Tookey J., Rotimi F.E. (2025). A SWOT analysis of stakeholder perspectives on the strategic application of economic sustainability indicators in Ghana’s road infrastructure development. Int. J. Sustain. Transp..

[29] Jelti F., Saadani R. (2024). Energy efficiency analysis of heavy goods vehicles in road transportation: The case of Morocco. Case Stud. Transp. Policy.

[30] European Parliament and Council (2008). Directive 2008/98/EC on Waste and Repealing Certain Directives.

[31] European Parliament and Council (2018). Directive (EU) 2018/851 Amending Directive 2008/98/EC on Waste.

[32] (2021). Unbound and Hydraulically Bound Mixtures—Part 47: Test Method for the Determination of California Bearing Ratio, Immediate Bearing Index and Linear Swelling.

[33] Judycki J., Jaskuła P., Pszczoła M., Ryś D., Jaczewski M., Alenowicz J., Dołżycki B., Stienss M. (2014). Katalog Typowych Konstrukcji Nawierzchni Podatnych i Półsztywnych.

[34] Sybilski D., Bańkowski W., Maliszewski M., Maliszewska D., Zofka A. (2013). Katalog Przebudów i Remontów Nawierzchni Podatnych i Półsztywnych.

[35] (2002). System Oceny Stanu Nawierzchni. Wytyczne Techniczne. [System of the Assessment of Pavement Condition. Technical Guidelines].

[36] (2016). Bituminous Mixtures—Material Specifications—Part 1: Asphalt Concrete.

[37] (2018). Unbound Mixtures—Specifications.

[38] (2002). Aggregates for Unbound and Hydraulically Bound Materials for Use in Civil Engineering Work and Road Construction.

[39] (2009). Bitumen and Bituminous Binders. Specifications for Paving Grade Bitumens.

[40] (2011). Cement—Part 1: Composition, Specifications and Conformity Criteria for Common Cements.

[41] (2016). Bituminous Mixtures—Material Specifications—Part 8: Reclaimed Asphalt.

[42] (2012). Tests for Geometrical Properties of Aggregates—Part 1: Determination of Particle Size Distribution—Sieving Method.

[43] (2020). Bituminous Mixtures—Test Methods—Part 1: Soluble Binder Content.

[44] (2013). Bituminous Mixtures—Test Methods—Part 3: Bitumen Recovery: Rotary Evaporator.

[45] (2024). Bitumen and Bituminous Binders—Determination of Needle Penetration.

[46] (2015). Bitumen and Bituminous Binders—Determination of the Softening Point—Ring and Ball Method.

[47] (2010). Unbound and Hydraulically Bound Mixtures—Part 2: Test Methods for Laboratory Dry Density and Water Content—Proctor Compaction.

[48] (2017). Bituminous Mixtures—Test Methods—Part 23: Determination of the Indirect Tensile Strength of Bituminous Specimens.

[49] (2004). Wirtgen Cold Recycling Technology. Manual.

[50] (2018). Bituminous Mixtures—Test Methods—Part 5: Determination of the Maximum Density.

[51] (2020). Bituminous Mixtures—Test Methods—Part 6: Determination of Bulk Density of Bituminous Specimens.

[52] (2018). Bituminous mixtures—Test Methods—Part 8: Determination of Void Characteristics of Bituminous Specimens.

[53] (2021). Unbound and Hydraulically Bound Mixtures; Part 41: Test Method for Determination of the Compressive Strength of Hydraulically Bound Mixtures.

[54] (2004). Unbound and Hydraulically Bound Mixtures—Part 50: Method for the Manufacture of Test Specimens of Hydraulically Bound Mixtures Using Proctor Equipment or Vibrating Table Compaction.

[55] (2022). Bituminous Mixtures—Test Methods—Part 26: Stiffness.

[56] (2018). Bituminous Mixtures—Test Methods—Part 30: Specimen Preparation by Impact Compactor.

[57] Eurostat, S.O. of the E.U. Glossary: Tonne-Kilometre (t-km). https://ec.europa.eu/eurostat/statistics-explained/index.php/Glossary:Tonne-kilometre_(tkm).

[58] Organisation for Economic Co-operation and Development (OECD) Glossary of Statistical Terms: Tonne-Kilometre (tkm) and Vehicle-Kilometre (vkm). https://www.oecd.org/en/publications/oecd-glossary-of-statistical-terms_9789264055087-en.html.

[59] (2025). Ecoinvent Database v3.12—Documentation of Changes Implemented.

[60] (2025). Circular Ecology. ICE Database v4.1—Embodied Carbon and Energy Coefficients for Materials.

[61] (2006). Environmental Management—Life Cycle Assessment—Principles and Framework.

[62] (2006). Environmental Management—Life Cycle Assessment—Requirements and Guidelines.

[63] National Cooperative Highway Research Program (2004). Guide for Mechanistic–Empirical Design of New and Rehabilitated Pavement Structures.

[64] (2020). FDM 14-10 Pavement Design.

[65] Judycki J., Gierasimiuk P. (2019). Analizy i Projektowanie Konstrukcji Nawierzchni Podatnych i Półsztywnych [Analyses and Design of Flexible and Semi-Rigid Pavement Structures].

[66] (2014). Nawierzchnie Asfaltowe na Drogach Krajowych. WT-2 2014—Część I. Mieszanki Mineralno-Asfaltowe. Wymagania Techniczne.

[67] (2010). Mieszanki Niezwiązane do Dróg Krajowych. Wymagania Techniczne WT-4.

[68] (2022). Tests for Mechanical and Physical Properties of Aggregates—Part 6: Determination of Particle Density and Water Absorption.

[69] (2012). Tests for Geometrical Properties of Aggregates—Part 3: Determination of Particle Shape—Flakiness Index.

[70] (2008). Tests for Geometrical Properties of Aggregates—Part 4: Determination of Particle Shape—Shape Index.

[71] (2020). Tests for Mechanical and Physical Properties of Aggregates—Part 2: Methods for the Determination of Resistance to Fragmentation.

[72] (2007). Tests for Thermal and Weathering Properties of Aggregates—Part 1: Determination of Resistance to Freezing and Thawing.

[73] Tarsi G., Tataranni P., Sangiorgi C. (2020). The Challenges of Using Reclaimed Asphalt Pavement for New Asphalt Mixtures: A Review. Materials.

[74] (2012). Wirtgen Cold Recycling Technology. Manual.

[75] Iwański M., Chomicz-Kowalska A., Mazurek G., Buczyński P., Iwański M.M., Maciejewski K., Ramiączek P. (2018). Procedury Projektowania Oraz Wytyczne Stosowania Materiałów Odpadowych i z Recyklingu do Technologii Wytwarzania Mieszanek Metodą na Zimno z Asfaltem Spienionym MCAS.

[76] Chen X., Wang H. (2018). Life cycle assessment of asphalt pavement recycling for greenhouse gas emission with temporal aspect. J. Clean. Prod..

[77] Giani M.I., Dotelli G., Brandini N., Zampori L. (2015). Comparative life-cycle assessment of asphalt pavements using reclaimed asphalt, warm-mix technology and cold in-place recycling. Resour. Conserv. Recycl..

[78] Harvey J., Kendall A., Santero N., Wang T. (2016). FHWA-HIF-16-014; Pavement Life Cycle Assessment Framework.

[79] UK Department for Energy Security and Net Zero (2023). Greenhouse Gas Reporting: Conversion Factors 2023 (GHG Conversion Factors for Company Reporting; Includes Heavy Goods Vehicle (HGV) Transport Factors). https://www.gov.uk/government/publications/greenhouse-gas-reporting-conversion-factors-2023.

[80] Dal Ben M., Jenkins K.J. (2014). Performance of cold recycling materials with foamed bitumen and increasing percentage of reclaimed asphalt pavement. Road Mater. Pavement Des..

[81] Buczyński P., Šrámek J., Mazurek G. (2023). The Influence of Recycled Materials on Cold Mix with Foamed Bitumen Properties. Materials.

[82] Meneses J., Vasconcelos K., Kuchiishi K., Bernucci L. (2025). Dynamic Modulus Regression Models for Cold Recycled Asphalt Mixtures. Infrastructures.

[83] Huang W., Cao M., Xiao L., Li J., Zhu M. (2023). Experimental study on the fatigue performance of emulsified asphalt cold recycled mixtures. Constr. Build. Mater..

[84] Zhu H.W., Pan J.P. (2012). Laboratory Experimental Research on Fatigue Performance of Cold Recycled Mixture. Appl. Mech. Mater..

[85] Carvajal M.E., Piratheepan M., Sebaaly P.E., Hajj E.Y., Hand A.J. (2021). Structural Contribution of Cold In-Place Recycling Base Layer. CivilEng.

[86] Sebaaly P.E., Bazi G., Hitti E., Weitzel D., Bemanian S. (2004). Performance of Cold In-Place Recycling in Nevada. Transp. Res. Rec. J. Transp. Res. Board.

[87] Santos J., Flintsch G., Ferreira A. (2017). Environmental and economic assessment of pavement construction and management practices for enhancing pavement sustainability. Resour. Conserv. Recycl..

[88] Gruber M.R., Hofko B. (2023). Life Cycle Assessment of Greenhouse Gas Emissions from Recycled Asphalt Pavement Production. Sustainability.

[89] Elaskary M.B., Awed A.M., Gabr A.R., El-Badawy S.M. (2024). Laboratory Characteristics, Performance Evaluation, and Agency Cost Analysis of Cold-Recycled Asphalt Mixtures. Mansoura Eng. J..

[90] Zaumanis M., Mallick R.B. (2015). Review of very high-content reclaimed asphalt use in plant-produced pavements: State of the art. Int. J. Pavement Eng..

[91] Sukhija M., Coleri E. (2025). A systematic review on the role of reclaimed asphalt pavement materials: Insights into performance and sustainability. Clean. Mater..

[92] Abdelaziz A., Ho C.H., Shan J., Almonnieay A. (2019). Effect of freeze-thaw cycles on fatigue cracking and rutting of asphalt pavements. Pavement and Asset Management.

